# Marine-Derived *Bacillus* and Their Potential as Probiotics

**DOI:** 10.3390/ijms27104352

**Published:** 2026-05-13

**Authors:** Natasha B. Bambridge, Yaoying Lu, Horst Joachim Schirra, Yunjiang Feng

**Affiliations:** 1 Institute for Biomedicine and Glycomics, Griffith University, Parklands Drive, Gold Coast, QLD 4222, Australia; tash.bambridge@griffithuni.edu.au (N.B.B.); y.lu@griffith.edu.au (Y.L.); h.schirra@griffith.edu.au (H.J.S.); 2 School of Environment and Science, Griffith University, 170 Kessels Road, Brisbane, QLD 4111, Australia

**Keywords:** *Bacillus*, marine-derived microbe, probiotics, safety, gut survivability, enzyme production

## Abstract

The marine environment is an underutilised resource in probiotic research despite its potential for unique and beneficial microbes. Bacterial probionts derived from the ocean are emerging in the probiotic research field as an area of interest. *Bacillus* species (spp.) are Gram-positive, endospore-forming bacteria. Due to their unique resilience and their generally recognised as safe (GRAS) status, they have gained traction as putative probiotics. Existing large-scale reviews into the probiotic potential of *Bacillus* spp. have focused on terrestrial species, with limited attention given to marine-derived species. This review aims to address this gap by evaluating marine-derived *Bacillus* spp. with a focus on their diversity, origins, sources and demonstrated potential as probionts.

## 1. Introduction

The marine environment is composed of many ecosystems, which are habitats to a variety of uniquely adapted organisms, including microbes [1,2,3]. Marine-derived microbes, specifically bacteria, have many useful applications, including as potential probiotics [2,4]. Probiotics, as defined by the World Health Organisation (WHO), are “live microorganisms that, when administered in adequate amounts, confer a health benefit on the host” [5,6]. In recent years, advancements in probiotic research have been motivated by antibiotic resistance, a public health threat observed in clinical isolates of bacteria that has been reported to have caused 1.14 million deaths in 2021 [7]. Probiotics present an alternative to antibiotics by preventing illness through a proactive approach [8]. This is achieved through a multi-mechanism approach involving pathogen modulation, immunity boosting and improved digestion, with the main result being an improvement to the animal’s health [8].

To be considered a probiotic: a microbe must meet certain essential criteria [4]. These include the microbe being non-pathogenic, non-resistant to antibiotics and able to survive and colonise the extreme environment of the digestive system [4,9]. Another desirable trait of a potential probiotic is the ability to confer benefits to the host, including immune and digestive enzyme production, pathogen mediation, and immune system modulation [4]. The most common form of microorganism utilised as probiotics is bacteria [6]. Lactic acid bacteria, namely, *Lactobacillus* and *Bifidobacterium*, are currently the most utilised probiotics as they originate from food fermentation or within the human and animal gut microbiomes [6,8,10,11,12]. Despite their wide usage, these bacteria are not optimal probiotics as they are easily impaired by the gastrointestinal (GI) tract and have little capability to colonise the gut [8,10,12]. Recently, investigations have focused on other bacterial families, including species from the genus *Bacillus* [8,11,12,13,14,15]. *Bacillus* is a Gram-positive, endospore-forming bacterial genus within the larger *Firmicutes* phylum [8,10,11,12,14,16]. The increased interest in *Bacillus* spp. is linked to their better storage properties and capability to survive in the gut [6,8,16,17]. *Bacillus* spp. is generally safe for human use, shows limited antibiotic resistance and promotes many significant health benefits [8].

While there have been reviews investigating *Bacillus* as a probiotic previously, these reviews have focused on terrestrial species [1,8,10,11,14,15,17,18]. This article aims to fill that gap by examining the present research on marine-derived *Bacillus* species in the past five years, with a specific emphasis on their applications to the probiotic field.

Two databases were utilised in this research: SCOPUS and ScienceDirect. A literature search of these two databases, using the search term “Marine-derived” and “*Bacillus*” with the inclusion criteria of peer-reviewed, English-only and journal articles, yielded 2025 results. This number was reduced to 1309 by restricting publication years to 2020 to 2025. The search was further refined to include articles in which “*Bacillus*” appeared in the title or abstract, while excluding articles that included “*Lactobacillus*” and “*Paenibacillus*” or “*Tubercle Bacillus*”, resulting in 723 papers. Articles focused on terrestrial *Bacillus* or reclassified as non-*Bacillus* were also eliminated. This resulted in 178 articles being included in this review, with an additional 39 articles cited for Supporting Information when the information could not be sourced from the original articles.

## 2. Marine-Derived *Bacillus* Diversity

### 2.1. Presence of Marine-Derived Bacillus Species and Their Associated Strains

*Bacillus* is a diverse genus of bacteria with hundreds of different taxonomically unique species. Within the literature, there were 33 different identified species and 40 unidentified species of *Bacillus* with a total of 525 strains reported (Table S1). Out of these strains, 17.3% (91) were identified as putative probiotics. *Bacillus cereus* was the most prevalent species identified in the literature: 38.4% (197) of all reported marine-derived isolates, but only 12.4% (11) were identified as putative probiotics (Table 1). *B. subtilis* encompassed 18.5% (97) of all reported marine-derived strains. However, 27.0% (24) were identified in the literature as probiotics, making it the most prevalent species in that field. Other commonly researched species, including *B. velezensis* (27), *B. licheniformis* (27), *B. pumilus* (20), and *B. amyloliquefaciens* (19), exhibited probiotic potential in 8, 10, 3 and 8 strains, respectively. 

### 2.2. Geographic and Oceanic Origins

Overall, within the literature, 33 countries were identified as the origins of marine-derived *Bacillus*, with 514 strains naming a specific country (Table S1). Asia is the largest contributor to the research, with 88% of all reported marine-derived *Bacillus* spp. and 86.5% of probiotics originating from Asia (Table 2). China is the origin of 54.7% (287) of these marine-derived strains and 62.4% (197) originated from the Paracel Islands within the South China Sea [19]. India was the origin of 16.4% (86) and the other 19.3% (89) originated from various Asian countries. In terms of probiotics, China was the origin of 44.9% (40) of putative probiotics, while India produced 23.6% (21).

Regarding oceanographic locations, the Pacific and India Oceans were the origins of 54.9% (288) and 21.3% (112) of all reported marine-derived *Bacillus* within the literature (Table 2). The South China Sea was the origin for 45.3% (238) of all literature isolates and 82.6% of all Pacific Ocean isolates. The Indian Ocean had a wider diversity of sea sources, with 25.9% (29) originating from the Bay of Bengal, 20.5% (23) from the Persian Gulf, 17.9% (20) from the Laccadive Sea and 13.4% from the Arabian Sea (15). In terms of probiotics, there was an even distribution (18) between the Indian and Pacific Oceans, with no other oceans contributing to this research (Table 2). Most of the probiotics were from the South China Sea (7) and the Bay of Bengal (11).

### 2.3. Sources

The marine environment is a thriving ecosystem consisting of water, sediments and an abundant flora and fauna species. Almost half of the *Bacillus* bacterial isolates identified in the literature originated from seawater (24.0%) or sediments (22.9%) (Table 3 and Table S1). However, other important sources of marine-derived *Bacillus* are corals (11.2%), plants (8.6%), algae (7.6%), fish (7.0%) and sponges (6.5%). Several *Bacillus* species, including *B. firmus* [20], *B subtilis* [21], *B. licheniformis* [21], *B. pumilus* [21], *B. cereus* [21], and *B. siamensis* [1,22], were isolated from different coral species. These included *Anthogorgia Caerulea* [1,22], *Scleractinia* spp. [23], *Acropora digitifera* [24], *Palythoa* spp. [21], and *Leptogorgia rigida* [20].

While water and sediments are the key sources of *Bacillus* spp., there are no probiotics isolated from seawater, and only seven are isolated from sediments (Table 3). Fish (34.8%) and aquaculture (23.6%) are the most common probiotic sources. Of all the strains isolated from these sources, 83.9% and 95.5% are used as probiotics, respectively. A variety of fish have been utilised to source probiotics, including: *Epinephalus* sp. (21.6%) [25,26,27,28,29], *Dicentrarchus* sp. (16.2%) [30,31,32], *Lates calcarifer* (8.1%) [33,34] and *Scopthalmus maximus* (8.1%) [35,36,37]. *Centroscyllium fabricii*, the black dogfish [38], and *Chiloscyllium plagiosum*, the white bamboo shark [39,40], were also used to isolate *B. amyloliquefaciens* strains BTS33 and GB-9. Isolates from invertebrates have also demonstrated probiotic potential (10.1%). Specifically, *B. baekryungensis* MS1 [41], *B. licheniformis* XW15, *B. subtilis* ZF3 and *B. subtilis* DB1 [42] isolated from a sediment in an *Apostichopus japonicus* aquaculture tank all displayed probiotic effects. One probiotic has been identified in coral, *B. tequilensis* Bt-Co [43], four in plants, none in algae and two in sponges. All the plant-related probiotics were isolated from mangroves despite seagrass being the main source of marine-derived *Bacillus* [19,44,45,46].

## 3. Marine-Derived *Bacillus* as Potential Probionts

**Figure 1:**
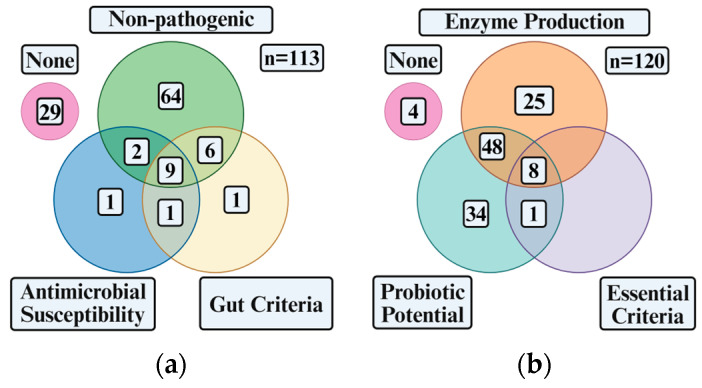
Marine-derived *Bacillus* assessment criteria: (**a**) Venn diagram of the literature-reported marine-derived *Bacillus* strains assessed against essential probiotic criteria, categorised by “Non-pathogenicity”, “Antimicrobial susceptibility”, and “Gut Criteria” (survivability and colonisation); strains failing all assessments are denoted as “None”. (**b**) Venn diagram of marine-derived *Bacillus* strains evaluated for “Enzyme Production”, “Essential Criteria” and “Probiotic Potential”.

As a necessity, any potential probiotic microbes must meet several criteria involving safety and function [4,9,47]. Safety is determined by eliminating potentially pathogenic microbes and those which may contribute to an increase in antibiotic resistance genes (ARG) [4,9,47]. Functionality is assessed by assessing a putative probiotic’s capability to survive in and colonise the gastrointestinal (GI) system [4,9,47]. Additional desirable criteria like immunomodulation or digestion are assessed through enzyme activity [4,9,47]. While there are 525 strains reported, only 21.5% (113) were assessed for a minimum of one essential probiotic criterion (Figure 1a). Only 17.3% (91) of these strains were identified as potential probiotics. Of the 91 strains, 9.9% (9) have met all the essential criteria (Figure 1a, Table 4): these strains are non-pathogenic, not antibiotic resistant and have demonstrated gut survivability. Within those that have met all the essential criteria, 88.9% (8) have been tested for enzyme production (Figure 1b, Table 4).

Only one, SYNSEA, a *Lactobacillus* and *B. subtilis* consortium from an unspecified fish [48], has been commercialised. The other eight *Bacillus* spp. have significant potential to be utilised as commercial probiotics (Table 4). Additional strains partially met the probiotic criteria, as shown in Table 5.

### 3.1. Investigations into Pathogenicity

*Bacillus* spp. have the potential to be pathogenic, especially strains like *B. cereus* and *B. anthracis* [49]. According to the European Food Safety Authority (EFSA), any putative probiotic *Bacillus* spp. must undergo in vivo testing to confirm safety or demonstrate that the cells are non-cytotoxic [50]. This is commonly evaluated through hemolytic activity assays. Bacterial species exhibiting β-hemolytic activity are considered pathogenic due to their ability to lyse red blood cells, whereas those displaying γ-hemolytic activity are regarded as non-pathogenic [28]. For members of the *B. cereus* species, they must undergo whole genome sequencing (WGS) to eliminate the presence of enterotoxin-coding genes (TCG), including *nhe*, *hbl*, and *cytK*, and cereulide synthase (*ces*) [50]. Marine-derived *Bacillus* were evaluated for their potential pathogenic effects in 15 distinct species and 90 strains (Table 6 and Table S2). Out of the 90 strains, 88.8% (80) were classified as non-pathogenic, <1% (5) required further testing and <1% (5) were considered pathogenic. *B. cereus* CH, HB, and WH from farmed *A. japonicus* [9] and *B. aerius* from the Marchika Lagoon [49] demonstrated β-hemolytic activity on blood agar, and the *B. cereus* strains contained 143 (CH), 140 (HB), and 141 (WH) virulence factors (VF) [9]. *B. cereus* G1–11 had γ-hemolysis and no mortality in animal trials but possessed toxin-coding genes *nhe* and *hbl*, excluding it as a potential probiotic [25]. Meanwhile, *B. subtilis* subsp. *inaquosorum* BSXE-2102 also possessed the same phenotypic traits and a TCG (*hly*-*III)* [51]. Due to the status of *B. subtilis* as GRAS and no evidence of the gene in the phenotype of the bacteria, it is classified as non-pathogenic.

As the most prevalent species in the probiotic literature, *B. subtilis* accounted for 26.7% (23) of non-pathogenic strains. Strains ABP1 and ABP2 both demonstrated γ-hemolysis, increased survival rates and reduced inflammation when administered as putative probiotics to *Oncorhynchus mykiss* [31]. *B. subtilis* BSXE-2102 was also identified as a potential probiotic as it increased survival rates when administered as a feed probiotic to *Penaeus vannamei*, as well as protected the animals from *Vibrio parahaemolyticus* infection [52]. A genomic analysis revealed that the presence of certain VFs, which can be associated with pathogenicity, played a beneficial role in the microbe’s effectiveness as a probiotic [52]. Of the 50 VFs, several genes, including 1 *dltA*, 1 *fbpaA* and 1 *fliP*, are associated with cell adherence [52]. In pathogenic bacteria, these traits are associated with infection risks, but in probiotics, they aid in gut colonisation [52].

### 3.2. Analysis of Antibiotic Resistance

Antibiotic resistance is the key driving factor behind probiotic advancement, and yet only 10.9% (57) of all reported marine-derived *Bacillus* strains have been investigated for their resistance, and only 24.8% (13) of the investigated strains met the EFSA standard. The EFSA states that phenotypic antimicrobial susceptibility testing is sufficient for microorganisms that do not exhibit acquired resistance [50]. However, for microorganisms that are typically susceptible but show resistance in phenotypic assays, whole-genome sequencing (WGS) is required to examine the genome for novel antimicrobial-resistant genes (ARGs) [50]. If such ARGs are identified, the strain is not considered eligible for use as a probiotic [50]. Eight commonly used antibiotics (Table S3) are recommended by EFSA, representing different antibiotic families. Out of the 57 strains, *B. subtilis* BSXE-2102 subsp. *inaquosorum*, isolated from *Litopaneaus vannamei* aquaculture [53], and *Bacillus* sp. PM8313, isolated from wild *Pagrus major*, were the only microbes found to be susceptible to all the recommended drugs and deemed safe for use as probiotics [54]; six microbes, including *B. velezensis* PGSAK01, *B. subtilis* subsp. *stercoris* PGSAK05, *B. velezensis* PGSAK17, *B. subtilis* PGSAK1 [28], *Bacillus* sp. RCS1 [25] and *B. cereus* G1–11 [25], were tested against alternative antibiotics within the same drug family and passed the standard, while five isolates, including *B. subtilis* subsp. *inaquosorum* M1 [51], *B. licheniformis* Ba4 [26], *B. licheniformis* [55], *B. aryabhattai* NM1-A2 [56,57] and *B. safensis* SDG14 [49], were validated through genomic sequencing. An additional five isolates were considered borderline, missing chloramphenicol or clindamycin from the panel. Another 37 needed further testing due to missing antibiotics, or in the case of *B. cereus* RCS3, there was resistance to vancomycin and doxycycline, which require further genomic analysis [58].

### 3.3. Gut Criteria

For a microbe to be considered as a potential probiotic, it must remain viable in the digestive tract, with the desired concentrations of bacteria in the GI tract being between ≥10^6^ and ≥10^8^ CFU/g [59,60]. The concentration of probiotics within the gut is dependent on the microbe’s ability to survive in the harsh conditions of the GI system and upon its capacity to colonise the intestine [59]. To survive the gut, a bacterial candidate must demonstrate a high tolerance to low pH and exposure to bile [59]. For colonisation, it must demonstrate the ability to adhere to the intestinal mucosa [52,61]. Out of 525 marine-derived *Bacillus* strains, 10.5% (55) have been tested for the ability to survive the gut and 3.6% (19) were tested for their ability to adhere to the gut (Table 7).

#### 3.3.1. Gut Survivability

Out of the 55 strains tested against this criterion, 38.2% (21) demonstrated the ability to survive a pH ≤ 3 and a bile salt concentration of at least 0.3% (Table 7). Some isolates required further testing, 20% (11) were missing either a pH tolerance test or a bile tolerance test, while 41.8% (23) failed as they were unable to survive in these conditions. Currently, there is no universally accepted standard for pH tolerance. The acidity of gastric juices varies significantly, with human stomach acid reaching a pH as low as 1.5 [52,59]. Survival in low pH is vital for putative probiotics, so a microbe that is able to survive in a pH of 3 or lower is considered acceptable for use as a probiotic. Overall, 21 isolates could survive in pH ≤ 3, including species *B. pumilus* [62], *B. safensis* [49,63], *B. subtilis* [28,51], *B. velezensis* [28], *B. haynesii* [64], and *Bacillus* sp. RCS1 [65]. Only six of the isolates were isolated from sources unrelated to the animal GI tract, including *B. aerius* S-4, *B. altitudinus* S-5, *B. pumilus* G-1 [4], *B. inaquosorum* M1 [51], *B. haynesii* CD223 [64] and *B. tequilensis* Bt-CO [43]. The widest pH tolerance was observed in four isolates originating from the hybrid grouper [28]. *B velezensis* PGSAK01, *B. stercosis* PGSAKA05, *B. velezensis* PGSAK17 and *B. subtilis* PGSAK19 all had pH tolerance between 1 and 10 [28]. PGSAK01, PGSAK05 and PGSAK17 had higher growth rates between pH 4 and 9, while PGSAK19 had a higher growth rate between pH 2 and 9 [28]. All of the isolates had the best growth at a more neutral pH between 6 and 8, indicating potential survival in the duodenum. While the stomach’s low acidity can impact the bacteria’s survival, bile possesses detergent-like characteristics, which further complicates the viability of bacteria [59].

There is no standard test to determine bile tolerance, but the generally accepted procedure is to test the bacteria’s survival in 0.3% bile salts [15,52,59]. The strains *B.* sp. RCS1 and *B. cereus* RCS3, both isolated from *Rachycentron canadum*, tolerated 0.5% bile salts for 3 h with a survivability of 54.06 and 65.38%, respectively [58]. Another species, *B. velezensis* D-18, demonstrated tolerance to 10% *L. Calcarifer* bile for 1.5 h [66]. Other tests utilised to assess acidity and bile tolerance involve examining tolerance to simulated intestinal (SIJ) or gastric juices (SGJ). *B. subtilis* subsp. *inaquosorum* M1 showed a survival rate of 65.67% in SIJ [51], while *B. tequilensis* Bt-CO was able to survive with a rate of 94.37% in SIJ and 92.59% in SGJ. *Bacillus* sp. KRF was tested for its tolerances to SGJ and SIJ in both vegetative and spore form [54,67]. The vegetative cells had much lower survival rates, 4.74 ± 0.68% and 3.64 ± 0.42%, respectively, while the spores had survival rates of 66.85 ± 4.07 and 5.96 ± 0.24% [67].

Temperature is not always considered in assessing gut survival, but through storage, processing and delivery, probiotics are exposed to varied temperatures, which may affect their viability [59,60,68]. Thermotolerant organisms have a higher survival rate when exposed to these variations and thus are better for industry use as probiotics [60]. If a bacterium was unable to survive at temperatures associated with life, 30–40°C, then they were excluded as being probiotics. The temperature tolerance varied depending on the strain. The lowest tolerated temperate was 15°C from *Pagrus major*-derived *Bacillus* sp. PM8313 [54], while the highest tolerated temperature was 100°C for five isolates, two from *Rachycentron canadum* (*Bacillus* sp. RCS1 and *B. cereus* RCS3) [58,65] and three from the gut of the hybrid grouper (*B. velezensis* PGSAK01, *B. velezensis* PGSAK17 and *B. subtilis* PGSAK19) [28]. Sixty-five percentage had a tolerance between 30 and 50°C and an optimum temperature tolerance between 30 and 40°C.

#### 3.3.2. Gut Colonisation

Overall, 19 (<1%) isolates were investigated for some form of colonisation capacity (Table 7). Of these 17 (89.5%) demonstrated the potential to adhere to the gut, while two (10.5%) were unable to adhere to the gut. Similarly to survivability, there is no current standardised method for examining cell adhesion within the gut [61]. However, current methods involve assessing auto-aggregation abilities and hydrophobicity or direct adhesion to cell lines [52,61]. Good adhesion is observed when auto-aggregation is over 50% [69,70,71] and hydrophobicity is over 30% [70] or cell adhesion is over 5% [61,72]. The highest auto-aggregation percentages were observed in *B. cereus* G1–11, which initially had moderate adhesion (<30%) during the first 5 h but increased highly to 93.83% after 24 h [25]. Comparatively, *Bacillus* sp. RCS1 and *B. cereus* RCS2 had initial auto-aggregation (<40%) at 3 h but rose to 80.6 ± 0.13% at the 24 h mark [58]. G1–11 also had the highest hydrophobicity (>90%), indicating the ability to adhere to intestinal mucosa, while *B. pumilus* A97 isolated from *Trachinotus ovatus* had a hydrophobicity level of 45.05% with xylene, 46.63% with chloroform and 45.38% with ethyl acetate [62]. Some strains have demonstrated adherence to cell lines even when they have poor auto-aggregation. *B. safensis* SDG14 demonstrated low auto-aggregation (18.4%) but relatively high cell adherence to Hep-2 Cell lines (45.54%), which indicates that it does have potential as a probiotic [73].

### 3.4. Enzyme Production

Digestive enzymes released by putative probiotics include carbohydrases, lipases, proteases, and peptidases. Releasing these digestive enzymes can confer benefits by increasing digestion, improving intestinal health, and modulating the gut microbiome [8,11,74]. Out of the 525 species, 13.1% (69) were documented as releasing a digestive-related enzyme. The digestive enzymes of particular interest were amylases, proteases, and lipases, which were produced across 12 different identified species (Table 8). Cellulase was also observed in seven different species. *B. subtilis* strains exhibited the highest enzyme production, with carbohydrases observed in 18 strains, lipases in seven, proteases in 18 and cellulase in 10. Enzyme activity varied between species. For example, *B. subtilis* SMF1, *B. licheniformis* LMF1 and *B. siamensis* DL3 all showed significantly higher protease and lipase activities (*p* < 0.05) but no significant alteration in the amylase activity when applied at 1 × 10^9^ CFU/g [75]. Multi-strain combination enhanced enzyme modulation: a mixture of *B. amyloliquefaciens* BN06, *B subtilis* WN07 and *P. megaterium* CT03 (1:1:1 ratio) resulted in significantly higher α-amylase and protease activity (*p* < 0.05) [46].

Only 78.8% (46) of all marine-derived *Bacillus* strains were examined for antioxidant-related enzymes, with 75.0% demonstrating activity. Antioxidant enzyme activity was reported across 12 *Bacillus* species (Table 8). Superoxide dismutase (SOD) activities were examined in 29 strains, catalase (CAT) in 19, and other oxidoreductase enzymes in four. *B. subtilis* was the most investigated, with eight strains exhibiting SOD activity, five showing CAT and two expressing other oxidoreductases. In *B. subtilis* W2Z, an increased dosage of probionts elevated the SOD and CAT activities in the hemolymph and hepatopancreas, while significantly reducing malondialdehyde (MDA) level [76]. Another strain, *B. subtilis* AAHM01, followed a similar trend with the addition of an increase in the activity of oxidoreductase and glutathione peroxidase [34]. Collectively, these findings demonstrate that marine-derived *Bacillus* spp. have the capability to reduce oxidative stress and improve host health [34,76]. Other immune-related enzymes, including lysozyme (LZM), alkaline phosphatase (AKP), acid phosphatase (ACP), transferases, and additional innate immune-related (II) enzymes, were documented in 10 *Bacillus* species (Table 9). LZM, AKP and ACP were the most frequently reported, occurring in 22, 19 and 21 strains, respectively. Again, *B. subtilis* was the most researched species, with seven strains exhibiting LZM activity, five showing AKP activity, and six exhibiting ACP activity. LZM is a key enzyme in the innate immune response due to its innate ability to kill pathogens [77]. A study on *B. velezensis* D-18, a putative probiotic, found that the bacteria boosted the bactericidal effects by increasing the LZM and the nitric oxide activities in *Dicentrarchus Labrax* [77]. Enhanced phagocytotic abilities have also been observed in *B. cereus* LS2, attributed to increased ACP and AKP levels [78].

## 4. Conclusions and Discussion

**Figure 2:**
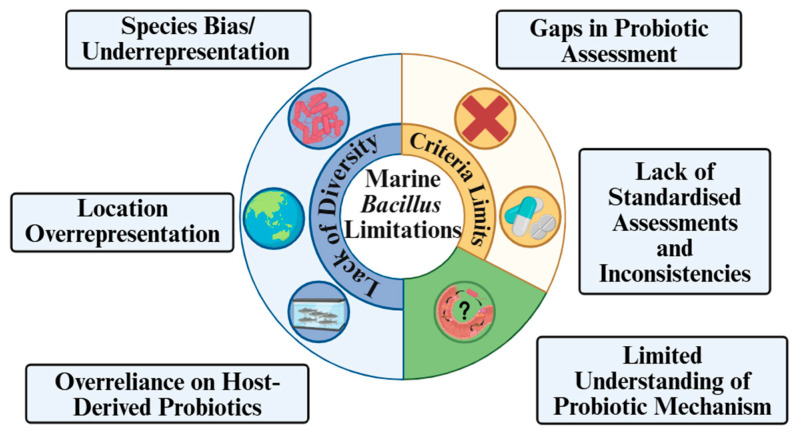
Overview of key limitations in current marine-derived *Bacillus* probiotic research, highlighting constraints related to “Lack of Diversity” (blue), “Criteria Limits” (yellow), and “Limited Understanding of Probiotic Mechanisms” (green).

The current review concludes that marine-derived *Bacillus* spp. isolates are predominately members of the *B. cereus* species, originating from China or the Pacific Ocean and typically sourced from seawater or sediments. Probiotic *Bacillus* strains are generally *B. subtilis*, also largely originating from China, and are more likely isolated from non-ocean marine sources such as farmed fish or aquaculture systems. Between 2020 and 2025, 525 marine-derived *Bacillus* strains were identified, yet only 91 strains were identified as probiotics. Of these 91 putative probiotic strains, only nine fulfilled all essential and desirable criteria required for potential commercialisation, and just one has been commercialised. An additional 33 non-probiotic strains met at least one of the criteria, highlighting the potential of marine-derived *Bacillus* spp. as probiotic candidates. Despite this promise, several key limitations are identified, which largely constrain the progress in the field (Figure 2).

### 4.1. Lack of Diversity

#### 4.1.1. Species Bias and Underrepresentation in Marine-Derived *Bacillus* Research

Of the 33 *Bacillus* species identified in the literature, less than half (45.5%, 15) have been investigated for their probiotic potential. Many of the unexamined species have very little research available, highlighting a clear knowledge gap, where these species may hold probiotic potential but have not been investigated beyond their original industry. Additionally, *B. cereus* is notably overrepresented in the current marine-derived *Bacillus* research, but only 0.06% (11) of isolates were identified as putative probiotics (Table 1). This is likely due to the potential pathogenicity associated with *B. cereus*, which limits its application in probiotic settings [9]. In contrast, *B. subtilis* was highly represented in probiotic research despite a moderate 27% success rate, largely due to the safety of *B. subtilis* [79]. However, other species show promising potential and may warrant greater attention. For instance, 50% of *B. safensis* and 40% of *B. pumilus* isolates were found to have probiotic characteristics. Further research into these species is recommended, as they may prove to be better probiotic candidates than *B. subtilis.* The need for further investigation is even more pronounced for less common species such as *B. australimaris* [80], *B. infantis* [16,81] and *B. stratosphericus* [1,82]. To date, only ten strains from these uncommon species have been investigated for their probiotic potential. Expanding the research base for these understudied species can help to identify species with superior probiotic capabilities and clarify the applications for which they are best suited.

#### 4.1.2. Location Overrepresentation and the Need for Broader Oceanic Exploration

Currently, China leads research on marine-derived *Bacillus* species (54.7%) and marine-derived probiotic research (44.9%) (Table 2). Consequently, most reported marine-derived *Bacillus* spp. originated from the South China Sea (45.3%), meaning they are sourced from similar environments and habitats. While investigating microhabitat variation is valuable for understanding how environmental origins can impact biological activities, there is a clear need to expand exploration into other oceanic locations, including the Arctic, Atlantic and Southern Oceans. These oceans have different ecosystems, which may yield microbes better suited for different industries and probiotic applications. The Southern and Arctic Oceans are particularly cold, which may create uniquely tolerate species to cold environments. Even investigations into different regions within the Pacific Ocean could potentially produce interesting *Bacillus* species with unique potential. Australia is surrounded by ecologically diverse seas, including the Coral, Timor, and Tasman Seas. Within the Coral Sea is the Great Barrier Reef, the world’s largest coral ecosystem, and an almost completely unexplored territory of unique probionts. Explorations into these untapped regions could lead to the discovery of novel *Bacillus* strains with probiotic potential.

#### 4.1.3. Overreliance on Host-Derived Strains in Probiotic Studies

While most of the marine-derived *Bacillus* strains originate from seawater, sediments and marine plants, these species are not being commonly utilised in probiotic research. Within probiotic research, more than half (57.2%) originated from farmed fish (34.1%) or the aquaculture environment (23.1%) (Table 2). This is connected to the overrepresentation of China, which contributes 44.0% (40) of the putative probiotic strains. It is common practice in aquaculture to use host-derived probiotics as they are convenient, effective and reduce costs associated with research. Gut-derived strains are ideal as they likely have an innate capacity to survive in the gut and reduce the risks associated with the introduction of microbes to the flock. However, this also means that species from more unconventional marine sources remain under-explored despite their potential value. Ocean-derived *Bacillus* represent a promising but largely untapped resource, as they often originate from extreme marine environments that confer unique physiological traits, including tolerance to varied temperatures, pH, and saline environments. Investigating these ocean-sourced *Bacillus* spp., both as food-supplemented and water additives, may yield uniquely tolerant bacteria.

Additionally, 97.8% of the putative probiotics were investigated in their efficacy in aquaculture, while only two studies investigated the effects of a marine-derived *Bacillus* species in goats [40] and pigs [83]. The focus on aquaculture is likely linked to their salinity tolerance, which makes them ideal candidates for this industry. However, limiting the species to aquaculture prevents the potential of the probiotics from being fully assessed. With the generally recognised safety of *Bacillus* species, further investigations into their application in agriculture across both livestock and plants could potentially yield valuable outcomes, as these environments also require hardy bacteria. Additionally, applications for humans are also a possibility once the essential probiotic criteria have been more stringently assessed and more evidence of safety within animals has been gathered.

### 4.2. Criteria Limitations

#### 4.2.1. Gaps in Probiotic Assessment of Marine-Derived *Bacillus*

Only 22.1% of the literature-reported marine-derived *Bacillus* have been evaluated for at least one essential probiotic criterion and just 17.3% were identified as putative probiotics. Of the 91 putative probiotic strains, only nine (Figure 1a) met all the essential criteria, with eight of these strains demonstrating enzyme-producing capabilities (Figure 1b). Most studies focused on identifying pathogenicity (75%) or enzyme production (70.8%). To date, only one commercial probiotic has utilised a marine-derived *Bacillus* species: SYNSEA, a mixture of *Lactobacillus* and *B. subtilis* isolated from fish and commercialised by SYNBIO Tech Inc. (Kaohsiung City, Taiwan) [48]. The fact that only one product has reached commercialisation out of 525 identified marine-derived *Bacillus* strains highlights the substantial untapped potential of marine-derived *Bacillus*. Given that only ten strains meet all the criteria, significant gaps remain to ensure that each putative probiotic is assessed against all the essential criteria. This is crucial to advance promising microbes to commercialisation. Beyond probiotic-focused research, there were also strains outside of the probiotics field which met at least one probiotic criterion. Of these, four were non-pathogenic, one met the EFSA standard, and 25 were assessed for enzyme activities. These isolates may represent promising candidates and warrant further investigation for their probiotic potential.

#### 4.2.2. Lack of Standardised Assessments and Inconsistencies

Despite the need for robustly defined criteria and standardised testing, the probiotic research field lacks clear standards for many criteria. Antimicrobial resistance was investigated in 57 studies, but only 13 strains met the EFSA standards. This indicates a significant gap in how antibiotic resistance is evaluated and accepted in potential probiotics. A key contributing factor is the absence of an international standard for determining whether to accept or reject a bacterial candidate based on its antibiotic resistance. Establishing such an international standard is challenging due to regional differences in antibiotic resistance. Currently, the most comprehensive standards are those provided by the EFSA and the CSLI standards. To ensure consistency in assessment, adopting the EFSA standards is an appropriate approach, as they are both comprehensive and freely available to researchers. Additionally, as genomic studies become more widely accessible, incorporating this process may assist in identifying ARGs prior to phenotypic testing. This approach could reduce the need for unnecessary antibiotics exposure and ensure that assessments more accurately reflect the genetic resistance potential of isolates.

Furthermore, methodologies and accepted standards related to bacterial survivability and colonisation vary significantly. When assessing hydrophobicity, the solvents used vary between studies and the cell lines used to examine adhesion are not consistent. For both pH, bile and adherence, there is a lack of defined standards, which creates a challenge when comparing studies. Due to variability in animal gut pH, researchers should clearly define their parameters for acceptance in their research. Regarding bile content, the current accepted tolerance is 0.3% bile salts and thus should be incorporated into research methods [15,52,59]. In terms of measuring probiotic colonisation capacity, auto-aggregation and hydrophobicity models are currently accepted and have been validated as appropriated methods [70]. However, better defining what constitutes a good result would ensure easier comparison between studies. When possible, observing adherence to Caco-2 cell lines with a comparative microbe may prove to be a better method as they simulate the GI tract more effectively [47,61]. Assessing the ability of a microbe to survive and colonise the GI tract is vital to determining whether it will be effective in vivo, so creating clear methods and standards is important to ensuring the quality of the probiotic product [59,60].

### 4.3. Limited Insights into Metabolite-Driven Probiotic Mechanisms

While probiotic research has extensively investigated the excretion of enzymes and their effects on the biological responses of host animals, limited research has been conducted on small-molecule metabolites excreted by these probiotics. *Bacillus* species are known to release metabolites with diverse bioactivities [1]. These metabolites may play a significant role in probiotic-associated health benefits. Although research has been conducted on host genomics and the microbiome, studies on host metabolomics remain limited. Metabolomics is important because, similarly to genomics, upregulations and downregulations of certain metabolites can provide insight into the mechanistic pathways through which probiotics exert their effects. Overall, a substantial gap remains in the chemical and mechanistic understanding of probiotic function. This gap could be filled through metabolite isolation and identification, paired with metabolomic research.

## Figures and Tables

**Table 1:** Count of marine-derived *Bacillus* strains reported within the literature between 2020 and 2025 and their applications.

*Bacillus* Strain	No. of Strains Used in Other Applications	No. of Strains Used as Probiotics
*B. altitudinus*	11	2
*B. amyloliquefaciens*	16	3
*B. cereus*	186	11
*B. firmus*	8	2
*B. licheniformis*	15	12
*B. pumilus*	12	8
*B. safensis*	10	5
*B. subtilis*	73	24
*B. velezensis*	19	8
Other *Bacillus* spp.	50	10
Unidentified *Bacillus* sp.	34	6

**Table 2:** Geographic and oceanographic origins of marine-derived *Bacillus* strains.

Geographic			Oceanographic		
Continent	No. of Strains Reported	No. of Probiotic Strains	Ocean	No. of Strains Reported	No. of Probiotic Strains
Africa	8	0	Arctic	2	0
Antarctica	1	0	Atlantic	27	0
Asia	462	79	Indian	112	20
China	287	40	Bay of Bengal	29	11
India	86	21	Laccadive Sea	20	0
Europe	20	7	Pacific	288	18
North America	11	5	South China Sea	238	7
South America	11	0	Southern	1	0
Oceania	1	0	Unspecified	1	0

**Table 3:** Source of marine-derived *Bacillus* strains.

Source	No. of Strains Reported	No. of Probiotic Strains	Source	No. of Strains Reported	No. of Probiotic Strains
Algae	38	2	Mollusks	5	3
Aquaculture	22	21	Plants	45	5
Collection	5	5	Seawater	126	0
Coral	59	1	Sediments	120	0
Crustacean	11	7	Sponges	36	2
Fish	37	31	Unknown	3	0
Invertebrates	16	9			

**Table 4:** List of the 9 marine-derived *Bacillus* strains which met all essential criteria as probiotics.

*Bacillus* Strains	Non-Pathogenic	Antimicrobial Susceptibility	Gut Criteria	Enzyme Production
*B. safensis* SDG14	✓	✓	✓	-
*Bacillus* sp. RCS1	✓	✓	✓	✓
*Bacillus* sp. PM8313	✓	✓	✓	✓
*B. velezensis* PGSAK01	✓	✓	✓	✓
*B. stercoris* PGSAK05	✓	✓	✓	✓
*B. velezensis* PGSAK17	✓	✓	✓	✓
*B. subtilis* PGSAK19	✓	✓	✓	✓
*B. subtilis* BSXE-2102	✓	✓	✓	✓
SYNSEA^TM^	✓	✓	✓	✓

✓: Criteria Met, -: Not Tested.

**Table 5:** Breakdown of marine-derived *Bacillus* strains against different probiotic criteria.

Probiotic Criteria	No. of Strains Used in Other Applications	No. of Strains Used as Probiotics
Non-pathogenic *Bacillus*	4	76
Antimicrobial Susceptible *Bacillus*	1	12
Gut Criteria	0	19
Enzyme Production	25	56
Total	433	91

**Table 6:** Marine-derived *Bacillus* species assessed for pathogenicity.

*Bacillus* Strains	Non-Pathogenic Species Reported as Probiotics	Non-Pathogenic Species Reported in Other Applications	Pathogenic Species
*B. aerius*	0	0	1
*B. altitudinus*	1	0	0
*B. amyloliquefaciens*	3	0	0
*B. baekryungensis*	1	0	0
*B. cereus*	7	0	4
*B. haynesii*	1	0	0
*B. firmus*	2	0	0
*B. licheniformis*	10	0	0
*B. pumilus*	6	0	0
*B. safensis*	5	0	0
*B. siamensis*	3	0	0
*B. subtilis*	21	2	0
*B. tequilensis*	1	0	0
*B. thuringiensis*	1	0	0
*B. velezensis*	8	2	0
Unidentified *Bacillus* sp.	6	0	0

**Table 7:** Marine-derived *Bacillus* tested for gut-related criteria.

Criteria	Met Criterion	Further Testing Required	Failed	Total Tested
Gut Survival	21	11	23	55
Gut Colonisation	17	2	0	19

**Table 8:** Distribution of species that produce digestive and antioxidant enzymes.

*Bacillus* Strains	Carbohydrases	Lipases	Proteases	Cellullase	SOD	CAT	Other
*B. altitudinus*	0	0	0	1	-	-	-
*B. amyloliquefaciens*	7	1	7	5	-	1	-
*B. baekryungensis*	-	-	-	-	1	1	1
*B. cereus*	8	5	6	4	4	3	2
*B. halotolerans*	4	1	2	3	-	-	-
*B. licheniformis*	3	5	3	3	3	3	0
*B. pumilus*	4	1	2	0	1	1	1
*B. safensis*	1	1	2	0	1	0	1
*B. siamensis*	0	1	1	0	1	0	0
*B. subtilis*	18	7	17	9	8	5	2
*B. tequilensis*	1	1	1	0	1	1	1
*B. thuringiensis*	1	1	1	0	1	1	1
*B. velezensis*	5	2	2	0	4	2	1
Unidentified *Bacillus* sp.	1	2	2	2	4	1	3

**Table 9:** Distribution of immune-related enzyme-producing capabilities by species.

*Bacillus* Species	LZM	ACP	AKP	Transferase	II
*B. baekryungensis*	0	1	1	0	1
*B. cereus*	1	2	3	0	1
*B. licheniformis*	5	3	3	0	0
*B. pumilus*	2	2	2	1	0
*B. safensis*	0	0	0	2	0
*B. siamensis*	1	1	1	2	0
*B. subtilis*	7	5	6	2	0
*B. tequilensis*	1	1	1	0	0
*B. velezensis*	3	4	4	0	0
Unidentified *Bacillus* sp.	2	0	0	0	0

## Data Availability

No new data were created or analysed in this study.

## Supplementary Materials

The following supporting information can be downloaded at: https://www.mdpi.com/article/10.3390/ijms27104352/s1.

## Abbreviations

The following abbreviations are used in this manuscript:

ACP	Acid Phosphatase
AKP	Alkaline Phosphatase
ARG	Antimicrobial-Resistant Gene
CAT	Catalase
CFU	Colony Forming Units
EFSA	European Food Safety Authority
GI	Gastrointestinal
GRAS	Generally Recognised as Safe
LZM	Lysozyme
SIJ	Simulated Intestinal Juices
SGJ	Simulated Gastric Juices
SOD	Superoxide Dismutase
VF	Virulence Factors
WGS	Whole Genome Sequencing
WHO	World Health Organisation
