# Supplementary material for: Marine-Derived Bacillus and Their Potential as Probiotics
Source: Int J Mol Sci. 2026 May 13;27(10):4352. doi: 10.3390/ijms27104352 (PMC13207364; doi:10.3390/ijms27104352)
Supplement: Supplementary file 1 [file ijms-27-04352-s001.zip › ijms-4224981-supplementary.pdf]

Supplementary information for

Marine *Bacillus* and their Potential as Probiotics

Natasha B. Bambridge [1], Yaoying Lu [1], Horst J. Schirra [1,2], and Yunjiang Feng [1,2,*]

[1] Institute for Biomedicine and Glycomics, Griffith University, Parklands Drive, Gold Coast, Queensland, 4222, Australia, 4111
[2] School of Environment and Science, Griffith University, 170 Kessels Road, Brisbane, Queensland, 4111, Australia
* Correspondence: y.feng@griffith.edu.au; Tel.: +61-(0)7-3735-8367

Academic Editor: First name Lastname

Received: date
Revised: date
Accepted: date
Published: date

Citation: To be added by editorial staff during production.

Copyright: © 2025 by the authors. Submitted for possible open access publication under the terms and conditions of the Creative Commons Attribution (CC BY) license (https://creativecommons.org/licenses/by/4.0/).

Contents

Supplementary Table S1- All Marine *Bacillus* between 2020-2025 3

Supplementary Table S2- Non-pathogenic Testing 23

Supplementary Table S4- Antibiotic Resistance 28

Supplementary Table S5- Gut Survivability 34

Supplementary Table S5- Gut Colonisation 41

Supplementary Table S6- Enzyme 45

Supplementary Table S1- All Marine *Bacillus* between 2020-2025

SPECIES	Strain	Probiotic	Country	Ocean	Sea	Source	REF
B. amyloliquefaciens	SCSIO 00856	✗	China	Pacific Ocean	South China Sea	Coral	[1]
Bacillus sp.	176	✗	China	Pacific Ocean	-	Sediments	[1]
B. stratosphericus	FLU5	✗	Tunisia	Atlantic Ocean	Mediterranean Sea	Seawater	[2]
B. velezensis	RP137	✗	Iran	Indian Ocean	Persian Gulf	Sediments	[3]
B. toyonensis	GAD1	✗	Egypt	Indian Ocean	Red Sea	Seawater	[3]
B. cereus	A30	✗	India	Indian Ocean	Palk Strait	Seawater	[3]
B. tequilensis	MS145	✗	India	Indian Ocean	Laccadive Sea	Sponge	[3]
B. baekryungensis	MS1	✓	China	-	-	Aquaculture	[4]
B. pumilus	A97	✓	China	-	-	Fish	[5]
B. cereus	MK966345	✓	India	-	-	Aquaculture	[6]
B. licheniformis	MK966367	✓	India	-	-	Aquaculture	[6]
B. subtilis	MK966350	✓	India	-	-	Aquaculture	[6]
Bacillus sp.	MK966347	✓	India	-	-	Aquaculture	[6]
B. subtilis	KC02	✗	Vietnam	Pacific Ocean	South China Sea	Sediments	[7]
B. subtilis	KC04	✗	Vietnam	Pacific Ocean	South China Sea	Sediments	[7]
B. subtilis	KC05	✗	Vietnam	Pacific Ocean	South China Sea	Sediments	[7]
B. subtilis	KC12	✗	Vietnam	Pacific Ocean	South China Sea	Sediments	[7]
B. subtilis	KC14	✗	Vietnam	Pacific Ocean	South China Sea	Sponge	[7]
B. subtilis	KC16	✗	Vietnam	Pacific Ocean	South China Sea	Sediments	[7]
B. subtilis	KC17	✗	Vietnam	Pacific Ocean	South China Sea	Sponge	[7]
B. subtilis	KC18	✗	Vietnam	Pacific Ocean	South China Sea	Algae	[7]
B. subtilis	KC20	✗	Vietnam	Pacific Ocean	South China Sea	Sponge	[7]
B. subtilis	KC22	✗	Vietnam	Pacific Ocean	South China Sea	Algae	[7]
B. amyloliquefaciens	KC06	✗	Vietnam	Pacific Ocean	South China Sea	Sponge	[7]
B. amyloliquefaciens	KCO8	✗	Vietnam	Pacific Ocean	South China Sea	Sponge	[7]

B. amyloliquefaciens	KC11	✗	Vietnam	Pacific Ocean	South China Sea	Algae	[7]
B. amyloliquefaciens[7]	KC13	✗	Vietnam	Pacific Ocean	South China Sea	Sediments	[7]
B. amyloliquefaciens	KC15	✗	Vietnam	Pacific Ocean	South China Sea	Sponge	[7]
B. licheniformis	KC03	✗	Vietnam	Pacific Ocean	South China Sea	Sediments	[7]
B. safensis	KC10	✗	Vietnam	Pacific Ocean	South China Sea	Algae	[7]
B. pacificus	KC07	✗	Vietnam	Pacific Ocean	South China Sea	Sponge	[7]
B. pacificus	KC09	✗	Vietnam	Pacific Ocean	South China Sea	Sponge	[7]
B. halotolerans	KC01	✗	Vietnam	Pacific Ocean	South China Sea	Sediments	[7]
B. halotolerans	KC19	✗	Vietnam	Pacific Ocean	South China Sea	Sponge	[7]
B. halotolerans	KC21	✗	Vietnam	Pacific Ocean	South China Sea	Algae	[7]
Bacillus sp.	B31	✗	China	Pacific Ocean	East China Sea	Sediments	[8]
B. licheniformis	Ge6-1	✓	China	-	-	Aquaculture	[9]
B. subtilis	0097	✗	Thailand	Pacific Ocean	Gulf of Thailand	Sponge	[10]
Bacillus sp.	-	✗	Canada	Arctic Ocean	-	Seawater	[11]
B. detrensis	-	✗	India	Indian Ocean	Andaman Sea	Seawater	[11]
B. licheniformis	AL 1.1	✗	Antarctica	Southern Ocean	Whaler's Bay	Sediment	[11]
B. subtilis	D5	✓	Saudi Arabia	-	-	Crustacean	[12]
Bacillus sp.	-	✗	China	Pacific Ocean	South China Sea	Sponge	[13]
Bacillus sp.	-	✗	India	Indian Ocean	Bay of Bengal	Sponge	[13]
Bacillus sp.	-	✗	-	Atlantic Ocean	Mediterranean Sea	Sponge	[13]
B. pumilus	B106	✗	China	Pacific Ocean	South China Sea	Sponge	[13]
B. velezensis	11-5	✗	China	Pacific Ocean	South China Sea	Sediments	[14, 15]
B. velezensis	SH-B74	✗	China	Pacific Ocean	South China Sea	Sediments	[16]
B. licheniformis	XW15	✓	China	-	-	Aquaculture	[17]
B. subtilis	ZF3	✓	China	-	-	Aquaculture	[17]
B. subtilis	DB1	✓	China	-	-	Aquaculture	[17]

Bacillus sp.	HC001	×	Republic of Korea	Pacific Ocean	Sea of Japan	Sediments	[18]
Bacillus sp.	4040	×	Canada	Pacific Ocean	Strait of Georgia	Sediments	[19]
B. pumilus	AMK1	×	India	Indian Ocean	Gulf of Mannar	Sponge	[20]
B. cereus	PB1-1	✓	Mexico	Pacific Ocean	-	Mollusc	[21]
B. firmus	PB106	✓	Mexico	Pacific Ocean	-	Mollusc	[21]
B. subtilis	A184	×	-	Atlantic Ocean	Mediterranean Sea	Sponge	[22]
B. velezensis	D-18	✓	Spain	-	-	Aquaculture	[23, 24]
B. licheniformis	09IDYM23	×	Republic of Korea	Pacific Ocean	Republic of Korea's Southern Reef	Sediments	[15, 25, 26]
Bacillus sp.	BC028	×	Germany	Atlantic Ocean	Baltic Sea	Mollusc	[15, 25]
B. licheniformis	BCR4-3	✓	Mexico	Pacific Ocean	Gulf Of California	Mollusc	[27]
B. subtilis	109GGC020	×	Republic of Korea	Pacific Ocean	Sea of Japan	Sediments	[25, 26, 28]
B. amyloliquefaciens	GB-9	✓	China	-	-	Fish	[29]
B. cereus	LS2	✓	China	-	-	Invertebrate	[30, 31]
B. pumilus	Sxm20-2	×	China	-	-	Sediments	[32]
B. pumilus	J33-1	×	China	-	-	Sediments	[32]
B. pumilus	C2-2	×	China	-	-	Sediments	[32]
B. pumilus	S8-t9-L9	×	China	-	-	Sediments	[32]
B. pumilus	DW2J2	×	China	-	-	Crustacean	[32]
B. pumilus	Sf214	×	China	-	-	Seawater	[32]
B. pumilus	RI06-95	×	China	-	-	Seawater	[32]
B. safensis	NH21E_2	×	China	-	-	Sediments	[32]
B. safensis	B204-B1-5	×	China	-	-	Sediments	[32]
B. safensis	D95	×	China	-	-	Sediments	[32]
B. safensis	15-B04-10-15-3	×	China	-	-	Sediments	[32]
B. safensis	NP-4	×	China	-	-	Seawater	[32]

B. safensis	S9	✗	China	-	-	Seawater	[32]
B. safensis	Fairview	✗	China	-	-	Seawater	[32]
B. altitudinus	S70-5-12	✗	China	-	-	Seawater	[32]
B. altitudinus	BS1	✗	China	-	-	Seawater	[32]
B. altitudinus	C16B11	✗	China	-	-	Seawater	[32]
B. altitudinus	C101	✗	China	-	-	Sediments	[32]
B. altitudinus	A23-8	✗	China	-	-	Sediments	[32]
B. altitudinus	Mn12	✗	China	-	-	Sediments	[32]
B. subtilis	-	✗	India	-	-	Crustacean	[33]
B. cereus	CH	✗	China	Pacific Ocean	Yellow Sea	Invertebrate	[34]
B. cereus	HB	✗	China	Pacific Ocean	Yellow Sea	Invertebrate	[34]
B. cereus	WH	✗	China	Pacific Ocean	Yellow Sea	Invertebrate	[34]
B. luti	-	✗	China	Pacific Ocean	Yellow Sea	Invertebrate	[34]
B. albus	-	✗	China	Pacific Ocean	Yellow Sea	Invertebrate	[34]
B. pumilus	AQP4275	✓	United Kingdom	-	-	Collection	[35]
B. toyonensis	BCT-7112T	✗	Mexico	Pacific Ocean	-	Crustacean	[36]
B. amyloliquefaciens	BN06	✓	India	Indian Ocean	Andaman Sea	Plant	[37]
B. subtilis	WN07	✓	India	Indian Ocean	Bay of Bengal	Plant	[37]
B. subtilis	MBTDCMFR IBa37	✓	India	Indian Ocean	Laccadive Sea	Sediments	[38]
B. licheniformis	MTCC 5514	✗	Italy	Atlantic Ocean	Tyrrhenian Sea	Seawater	[39]
B. thuringiensis	QQ1	✓	Iran	Indian Ocean	Persian Gulf	Fish	[40, 41]
B. cereus	QQ2	✓	Iran	Indian Ocean	Persian Gulf	Fish	[40, 41]
B. altitudinus	19_A	✗	Colombia	-	-	Algae	[42, 43]
B. safensis	SDG14	✓	India	-	-	Fish	[44]
Bacillus sp.	SCSIO 15121	✗	India	Indian Ocean	-	Sponge	[45]
Bacillus sp.	-	✗	India	Indian Ocean	-	Seawater	[45]

B. subtilis	C5	✗	China	Pacific Ocean	Yellow Sea	Sediments	[45]
Bacillus sp.	-	✗	India	Indian Ocean	Bay of Bengal	Sediments	[45]
B. subtilis	KC433737	✗	India	Indian Ocean	Gulf of Mannar	Seawater	[26]
B. velezensis	H3	✗	China	Pacific Ocean	Yellow Sea	Sediments	[26]
B. amyloliquefaciens	Anti-Ca	✗	-	-	-	Plant	[26]
B. tequilensis	CH	✗	India	Indian Ocean	Bay of Bengal	Seawaters	[26]
B. licheniformis	NIOT-AMKV06	✗	India	Indian Ocean	Andaman sea	Sponge	[26]
B. siamensis	-	✗	China	-	-	Coral	[46]
B. safensis	SQVG18	✓	China	-	-	Crustacean	[47]
B. cereus	-	✗	Turkey	-	Sea of Marmara	Sponge	[48]
B. amyloliquefaciens	L1	✗	China	-	-	Fish	[49]
B. cereus	SN7	✗	Indonesia	Indian Ocean	Strait of Malacca	Seawater	[50]
B. velezensis	B-9987	✗	China	Pacific Ocean	Yellow Sea	Plant	[51]
B. firmus	BSCB-13	✓	India	-	-	Crustacean	[52]
B. safensis	VQV8	✓	Taiwan	-	-	Crustacean	[53]
B. tropicus	W1	✗	China	Pacific Ocean	South China Sea	Seawater	[54]
B. tropicus	S1	✗	China	Pacific Ocean	South China Sea	Sediments	[54]
B. paramycoides	C1	✗	China	Pacific Ocean	South China Sea	Coral	[54]
B. paramycoides	C2	✗	China	Pacific Ocean	South China Sea	Coral	[54]
B. paramycoides	C3	✗	China	Pacific Ocean	South China Sea	Coral	[54]
B. tropicus	W2	✗	China	Pacific Ocean	South China Sea	Seawater	[54]
B. paramycoides	W3	✗	China	Pacific Ocean	South China Sea	Seawater	[54]
B. tropicus	W4	✗	China	Pacific Ocean	South China Sea	Seawater	[54]
B. tropicus	W5	✗	China	Pacific Ocean	South China Sea	Seawater	[54]
B. paramycoides	W6	✗	China	Pacific Ocean	South China Sea	Seawater	[54]
B. paramycoides	W7	✗	China	Pacific Ocean	South China Sea	Seawater	[54]
B. paramycoides	W8	✗	China	Pacific Ocean	South China Sea	Seawater	[54]

B. paramycoides	W9	×	China	Pacific Ocean	South China Sea	Seawater	[54]
B. tropicus	W10	×	China	Pacific Ocean	South China Sea	Seawater	[54]
B. paramycoides	S2	×	China	Pacific Ocean	South China Sea	Sediments	[54]
B. paramycoides	S3	×	China	Pacific Ocean	South China Sea	Sediments	[54]
B. paramycoides	S4	×	China	Pacific Ocean	South China Sea	Sediments	[54]
B. paramycoides	S5	×	China	Pacific Ocean	South China Sea	Sediments	[54]
B. paramycoides	W11	×	China	Pacific Ocean	South China Sea	Seawater	[54]
B. paramycoides	W12	×	China	Pacific Ocean	South China Sea	Seawater	[54]
B. paramycoides	W13	×	China	Pacific Ocean	South China Sea	Seawater	[54]
B. cereus	W14	×	China	Pacific Ocean	South China Sea	Seawater	[54]
B. paramycoides	W15	×	China	Pacific Ocean	South China Sea	Seawater	[54]
B. altitudinus	S7	×	China	Pacific Ocean	South China Sea	Sediments	[54]
B. cereus	S8	×	China	Pacific Ocean	South China Sea	Sediments	[54]
B. cereus	S9	×	China	Pacific Ocean	South China Sea	Sediments	[54]
B. tropicus	W16	×	China	Pacific Ocean	South China Sea	Seawater	[54]
B. paramycoides	S10	×	China	Pacific Ocean	South China Sea	Sediments	[54]
B. paramycoides	S11	×	China	Pacific Ocean	South China Sea	Sediments	[54]
B. tropicus	G1	×	China	Pacific Ocean	South China Sea	Plant	[54]
B. paramycoides	G2	×	China	Pacific Ocean	South China Sea	Plant	[54]
B. paramycoides	G3	×	China	Pacific Ocean	South China Sea	Plant	[54]
B. paramycoides	G4	×	China	Pacific Ocean	South China Sea	Plant	[54]
B. tropicus	G5	×	China	Pacific Ocean	South China Sea	Plant	[54]
B. paramycoides	G6	×	China	Pacific Ocean	South China Sea	Plant	[54]
B. wiedmannii	W17	×	China	Pacific Ocean	South China Sea	Seawater	[54]
B. wiedmannii	W18	×	China	Pacific Ocean	South China Sea	Seawater	[54]
B. paramycoides	W19	×	China	Pacific Ocean	South China Sea	Seawater	[54]
B. paramycoides	W20	×	China	Pacific Ocean	South China Sea	Seawater	[54]
B. tropicus	W21	×	China	Pacific Ocean	South China Sea	Seawater	[54]

B. paramycoides	W22	×	China	Pacific Ocean	South China Sea	Seawater	[54]
B. paramycoides	W23	×	China	Pacific Ocean	South China Sea	Seawater	[54]
B. tropicus	S12	×	China	Pacific Ocean	South China Sea	Sediments	[54]
B. paramycoides	S13	×	China	Pacific Ocean	South China Sea	Sediments	[54]
B. paramycoides	C5	×	China	Pacific Ocean	South China Sea	Coral	[54]
B. tropicus	W24	×	China	Pacific Ocean	South China Sea	Seawater	[54]
B. tropicus	W25	×	China	Pacific Ocean	South China Sea	Seawater	[54]
B. firmus	C6	×	China	Pacific Ocean	South China Sea	Coral	[54]
B. paramycoides	C7	×	China	Pacific Ocean	South China Sea	Coral	[54]
B. paramycoides	S14	×	China	Pacific Ocean	South China Sea	Sediments	[54]
B. tropicus	S15	×	China	Pacific Ocean	South China Sea	Sediments	[54]
B. tropicus	S16	×	China	Pacific Ocean	South China Sea	Sediments	[54]
B. paramycoides	S17	×	China	Pacific Ocean	South China Sea	Sediments	[54]
B. paramycoides	S18	×	China	Pacific Ocean	South China Sea	Sediments	[54]
B. paramycoides	W26	×	China	Pacific Ocean	South China Sea	Seawater	[54]
B. tropicus	W28	×	China	Pacific Ocean	South China Sea	Seawater	[54]
B. wiedmannii	W29	×	China	Pacific Ocean	South China Sea	Seawater	[54]
B. tropicus	W30	×	China	Pacific Ocean	South China Sea	Seawater	[54]
B. tropicus	W31	×	China	Pacific Ocean	South China Sea	Seawater	[54]
B. paramycoides	W32	×	China	Pacific Ocean	South China Sea	Seawater	[54]
B. paramycoides	W33	×	China	Pacific Ocean	South China Sea	Seawater	[54]
B. paramycoides	W34	×	China	Pacific Ocean	South China Sea	Seawater	[54]
B. cereus	W35	×	China	Pacific Ocean	South China Sea	Seawater	[54]
B. paramycoides	W36	×	China	Pacific Ocean	South China Sea	Seawater	[54]
B. paramycoides	W37	×	China	Pacific Ocean	South China Sea	Seawater	[54]
B. paramycoides	W38	×	China	Pacific Ocean	South China Sea	Seawater	[54]
B. tropicus	W39	×	China	Pacific Ocean	South China Sea	Seawater	[54]
B. tropicus	W40	×	China	Pacific Ocean	South China Sea	Seawater	[54]

B. paramycoides	W41	×	China	Pacific Ocean	South China Sea	Seawater	[54]
B. paramycoides	W42	×	China	Pacific Ocean	South China Sea	Seawater	[54]
B. paramycoides	W43	×	China	Pacific Ocean	South China Sea	Seawater	[54]
B. tropicus	W44	×	China	Pacific Ocean	South China Sea	Seawater	[54]
B. tropicus	W45	×	China	Pacific Ocean	South China Sea	Seawater	[54]
B. paramycoides	W46	×	China	Pacific Ocean	South China Sea	Seawater	[54]
B. tropicus	W47	×	China	Pacific Ocean	South China Sea	Seawater	[54]
B. tropicus	W48	×	China	Pacific Ocean	South China Sea	Seawater	[54]
B. mycoides	W49	×	China	Pacific Ocean	South China Sea	Seawater	[54]
B. tropicus	W50	×	China	Pacific Ocean	South China Sea	Seawater	[54]
B. paramycoides	W51	×	China	Pacific Ocean	South China Sea	Seawater	[54]
B. tropicus	W52	×	China	Pacific Ocean	South China Sea	Seawater	[54]
B. tropicus	W53	×	China	Pacific Ocean	South China Sea	Seawater	[54]
B. paramycoides	W54	×	China	Pacific Ocean	South China Sea	Seawater	[54]
B. paramycoides	W56	×	China	Pacific Ocean	South China Sea	Seawater	[54]
B. paramycoides	W57	×	China	Pacific Ocean	South China Sea	Seawater	[54]
B. tropicus	W58	×	China	Pacific Ocean	South China Sea	Seawater	[54]
B. tropicus	W59	×	China	Pacific Ocean	South China Sea	Seawater	[54]
B. bingmayongensis	W61	×	China	Pacific Ocean	South China Sea	Seawater	[54]
B. paramycoides	W62	×	China	Pacific Ocean	South China Sea	Seawater	[54]
B. tropicus	W63	×	China	Pacific Ocean	South China Sea	Seawater	[54]
B. tropicus	S19	×	China	Pacific Ocean	South China Sea	Sediments	[54]
B. paramycoides	S20	×	China	Pacific Ocean	South China Sea	Sediments	[54]
B. aryabhattai	G8	×	China	Pacific Ocean	South China Sea	Plant	[54]
B. aryabhattai	G9	×	China	Pacific Ocean	South China Sea	Plant	[54]
B. zanthoxyli	G10	×	China	Pacific Ocean	South China Sea	Plant	[54]
B. aryabhattai	G11	×	China	Pacific Ocean	South China Sea	Plant	[54]
B. zanthoxyli	G12	×	China	Pacific Ocean	South China Sea	Plant	[54]

B. zanthoxyli	G14	×	China	Pacific Ocean	South China Sea	Plant	[54]
B. tropicus	S21	×	China	Pacific Ocean	South China Sea	Sediments	[54]
B. tropicus	W65	×	China	Pacific Ocean	South China Sea	Seawater	[54]
B. tropicus	S22	×	China	Pacific Ocean	South China Sea	Sediments	[54]
B. tropicus	S23	×	China	Pacific Ocean	South China Sea	Sediments	[54]
B. tropicus	S24	×	China	Pacific Ocean	South China Sea	Sediments	[54]
B. tropicus	S25	×	China	Pacific Ocean	South China Sea	Sediments	[54]
B. paramycoides	S26	×	China	Pacific Ocean	South China Sea	Sediments	[54]
B. paramycoides	C8	×	China	Pacific Ocean	South China Sea	Coral	[54]
B. paramycoides	C9	×	China	Pacific Ocean	South China Sea	Coral	[54]
B. paramycoides	W66	×	China	Pacific Ocean	South China Sea	Seawater	[54]
B. paramycoides	S27	×	China	Pacific Ocean	South China Sea	Sediments	[54]
B. paramycoides	S28	×	China	Pacific Ocean	South China Sea	Sediments	[54]
B. paramycoides	C10	×	China	Pacific Ocean	South China Sea	Coral	[54]
B. wiedmannii	C12	×	China	Pacific Ocean	South China Sea	Coral	[54]
B. paramycoides	W67	×	China	Pacific Ocean	South China Sea	Seawater	[54]
B. tropicus	W68	×	China	Pacific Ocean	South China Sea	Seawater	[54]
B. tropicus	W69	×	China	Pacific Ocean	South China Sea	Seawater	[54]
B. paramycoides	C13	×	China	Pacific Ocean	South China Sea	Coral	[54]
B. paramycoides	C14	×	China	Pacific Ocean	South China Sea	Coral	[54]
B. paramycoides	C15	×	China	Pacific Ocean	South China Sea	Coral	[54]
B. paramycoides	C16	×	China	Pacific Ocean	South China Sea	Coral	[54]
B. paramycoides	C17	×	China	Pacific Ocean	South China Sea	Coral	[54]
B. paramycoides	C18	×	China	Pacific Ocean	South China Sea	Coral	[54]
B. paramycoides	C19	×	China	Pacific Ocean	South China Sea	Coral	[54]
B. paramycoides	C20	×	China	Pacific Ocean	South China Sea	Coral	[54]
B. paramycoides	C21	×	China	Pacific Ocean	South China Sea	Coral	[54]
B. paramycoides	W70	×	China	Pacific Ocean	South China Sea	Seawater	[54]

B. tropicus	W71	✕	China	Pacific Ocean	South China Sea	Seawater	[54]
B. paramycoides	W72	✕	China	Pacific Ocean	South China Sea	Seawater	[54]
B. paramycoides	W73	✕	China	Pacific Ocean	South China Sea	Seawater	[54]
B. cereus	W74	✕	China	Pacific Ocean	South China Sea	Seawater	[54]
B. paramycoides	S29	✕	China	Pacific Ocean	South China Sea	Sediments	[54]
B. paramycoides	S30	✕	China	Pacific Ocean	South China Sea	Sediments	[54]
B. paramycoides	C22	✕	China	Pacific Ocean	South China Sea	Coral	[54]
B. paramycoides	C23	✕	China	Pacific Ocean	South China Sea	Coral	[54]
B. paramycoides	C24	✕	China	Pacific Ocean	South China Sea	Coral	[54]
B. paramycoides	W75	✕	China	Pacific Ocean	South China Sea	Seawater	[54]
B. paramycoides	S31	✕	China	Pacific Ocean	South China Sea	Sediment	[54]
B. paramycoides	S32	✕	China	Pacific Ocean	South China Sea	Sediments	[54]
B. paramycoides	S33	✕	China	Pacific Ocean	South China Sea	Sediments	[54]
B. paramycoides	S34	✕	China	Pacific Ocean	South China Sea	Sediments	[54]
B. paramycoides	C25	✕	China	Pacific Ocean	South China Sea	Coral	[54]
B. paramycoides	C26	✕	China	Pacific Ocean	South China Sea	Coral	[54]
B. paramycoides	C27	✕	China	Pacific Ocean	South China Sea	Coral	[54]
B. paramycoides	W76	✕	China	Pacific Ocean	South China Sea	Seawater	[54]
B. paramycoides	W77	✕	China	Pacific Ocean	South China Sea	Seawater	[54]
B. paramycoides	W78	✕	China	Pacific Ocean	South China Sea	Seawater	[54]
B. tropicus	W79	✕	China	Pacific Ocean	South China Sea	Seawater	[54]
B. paramycoides	S35	✕	China	Pacific Ocean	South China Sea	Sediments	[54]
B. paramycoides	S36	✕	China	Pacific Ocean	South China Sea	Sediments	[54]
B. paramycoides	S37	✕	China	Pacific Ocean	South China Sea	Sediments	[54]
B. paramycoides	C28	✕	China	Pacific Ocean	South China Sea	Coral	[54]
B. paramycoides	C29	✕	China	Pacific Ocean	South China Sea	Coral	[54]
B. paramycoides	C30	✕	China	Pacific Ocean	South China Sea	Coral	[54]
B. paramycoides	C31	✕	China	Pacific Ocean	South China Sea	Coral	[54]

B. paramycoides	C32	✗	China	Pacific Ocean	South China Sea	Coral	[54]
B. paramycoides	C33	✗	China	Pacific Ocean	South China Sea	Coral	[54]
B. paramycoides	C34	✗	China	Pacific Ocean	South China Sea	Coral	[54]
B. paramycoides	C35	✗	China	Pacific Ocean	South China Sea	Coral	[54]
B. paramycoides	C36	✗	China	Pacific Ocean	South China Sea	Coral	[54]
B. paramycoides	C37	✗	China	Pacific Ocean	South China Sea	Coral	[54]
B. cereus	W80	✗	China	Pacific Ocean	South China Sea	Seawater	[54]
B. tropicus	W81	✗	China	Pacific Ocean	South China Sea	Seawater	[54]
B. tropicus	W82	✗	China	Pacific Ocean	South China Sea	Seawater	[54]
B. tropicus	W83	✗	China	Pacific Ocean	South China Sea	Seawater	[54]
B. paramycoides	S38	✗	China	Pacific Ocean	South China Sea	Sediments	[54]
B. paramycoides	C39	✗	China	Pacific Ocean	South China Sea	Coral	[54]
B. paramycoides	C40	✗	China	Pacific Ocean	South China Sea	Coral	[54]
B. subtilis	C41	✗	China	Pacific Ocean	South China Sea	Coral	[54]
B. paramycoides	C42	✗	China	Pacific Ocean	South China Sea	Coral	[54]
B. paramycoides	C43	✗	China	Pacific Ocean	South China Sea	Coral	[54]
B. paramycoides	S39	✗	China	Pacific Ocean	South China Sea	Sediments	[54]
B. tropicus	S40	✗	China	Pacific Ocean	South China Sea	Sediments	[54]
B. aerius	S41	✗	China	Pacific Ocean	South China Sea	Sediments	[54]
B. paramycoides	C44	✗	China	Pacific Ocean	South China Sea	Coral	[54]
B. paramycoides	G18	✗	China	Pacific Ocean	South China Sea	Plant	[54]
B. paramycoides	G19	✗	China	Pacific Ocean	South China Sea	Plant	[54]
B. tropicus	G20	✗	China	Pacific Ocean	South China Sea	Plant	[54]
B. tropicus	G21	✗	China	Pacific Ocean	South China Sea	Plant	[54]
B. tropicus	G22	✗	China	Pacific Ocean	South China Sea	Plant	[54]
B. bingmayongensis	G23	✗	China	Pacific Ocean	South China Sea	Plant	[54]
B. bingmayongensis	G24	✗	China	Pacific Ocean	South China Sea	Plant	[54]
B. subtilis	SYNSEA	✓	Taiwan	-	-	Fish	[55]

B. pumilus	36R ATNSAL	✓	Mexico	Pacific Ocean	Gulf of California	Sediments	[56]
B. safensis	13L LOBSAL	✓	Mexico	Pacific Ocean	Gulf of California	Sediments	[56]
B. velezensis	MVCR2	✓	China	-	-	Fish	[57]
Bacillus sp.	SW1-1	✓	Republic of Korea	-	-	Crustacean	[58]
Bacillus spp.	-	✗	Taiwan	-	-	Coral	[59]
B. subtilis	S3L1	✓	India	Indian Ocean	Bay of Bengal	Invertebrate	[60]
B. subtilis	S3L4	✓	India	Indian Ocean	Bay of Bengal	Invertebrate	[60]
B. licheniformis	S3Z2A	✓	India	Indian Ocean	Bay of Bengal	Invertebrate	[60]
B. pumilus	S3L6	✓	India	Indian Ocean	Bay of Bengal	Invertebrate	[60]
B. halotolerans	S1L2	✓	India	Indian Ocean	Bay of Bengal	Invertebrate	[60]
B. cereus	S1L1	✓	India	Indian Ocean	Bay of Bengal	Invertebrate	[60]
B. cereus	S3Z2B	✓	India	Indian Ocean	Bay of Bengal	Invertebrate	[60]
B. cereus	S3A1	✓	India	Indian Ocean	Bay of Bengal	Invertebrate	[60]
B. subtilis	P2.24	✓	Indonesia	Pacific Ocean	Java Sea	Sponge	[61]
B. altitudinus	D6.19	✓	Indonesia	Pacific Ocean	Java Sea	Sponge	[61]
B. subtilis	FI314	✓	Portugal	-	-	Fish	[62]
B. velezensis	FI436	✓	Portugal	-	-	Fish	[62]
B. pumilus	FI464	✓	Portugal	-	-	Fish	[62]
B. subtilis	MMA7	✗	Ireland	-	-	Sponge	[63]
Bacillus spp.	-	✗	Ireland	-	-	Algae	[63]
B. paralicheniformis	APC 1576	✗	Ireland	-	-	Fish	[63]
B. subtilis	EP1	✗	Oman	Indian Ocean	Arabian Sea	Plant	[64]
B. safensis	SH10	✗	Oman	Indian Ocean	Arabian Sea	Plant	[64]
B. licheniformis	EI-34-6	✗	United Kingdom	-	-	Algae	[65]
Bacillus sp.	B1	✗	China	Pacific Ocean	South China Sea	Algae	[66]

Bacillus sp.	SY-1	×	Republic of Korea	Pacific Ocean	Sea of Japan	Seawater	[66]
B. subtilis	ABP1	✓	Spain	-	-	Fish	[67, 68]
B. subtilis	ABP2	✓	Spain	-	-	Fish	[67]
B. altitudinus	-	×	China	-	-	Algae	[69]
B. siamensis	168CLC-66.1	×	Vietnam	Pacific Ocean	South China Sea	Algae	[70]
B. subtilis	SY2101	×	-	Pacific Ocean	Philippine Sea	Sediments	[71]
B. cereus	UCP 1515	×	Brazil	Atlantic Ocean	-	Seawater	[72]
B. methylotrophicus	UCP 1615	×	Brazil	Atlantic Ocean	-	Seawater	[72]
B. amyloliquefaciens	MTCC 12713	×	India	Indian Ocean	Gulf of Mannar	Algae	[73]
B. licheniformis	PNB3	✓	China	-	-	Aquaculture	[74]
B. licheniformis	MB01	×	China	Pacific Ocean	Yellow Sea	Sediments	[25]
Bacillus sp.	SPB7	×	India	Indian Ocean	Bay of Bengal	Sponge	[25]
B. subtilis	MTCC 10407	×	India	Indian Ocean	Gulf of Mannar	Algae	[25]
B. amyloliquefaciens	-	×	India	Indian Ocean	Gulf of Mannar	Algae	[25]
B. subtilis	MTCC 10403	×	India	Indian Ocean	Gulf of Mannar	Algae	[25]
B. stratosphericus	16L088-2	×	India	Indian Ocean	Gulf of Mannar	Unspecified	[25]
B. pumilus	RJA 1515	×	Canada	Pacific Ocean	-	Sediments	[25]
B subtilis	AD35	×	Egypt	Atlantic Ocean	Alexandria Sea	Seawater	[3, 25]
B. amyloliquefaciens	SH-B74	×	China	Pacific Ocean	South China Sea	Sediments	[25]
B. mojavensis	B0621A	×	China	Pacific Ocean	Sea of Japan	Mollusc	[25]
Bacillus sp.	-	×	Korea	Pacific Ocean	Sea of Japan	Seawater	[25]
Bacillus sp.	UST050418-715	×	United State of America	Pacific Ocean	Salish Sea	Sponge	[25]
Bacillus sp.	-	×	India	Indian Ocean	Arabian Sea	Sponge	[25]
B. amyloliquefaciens	MTCC 10456	×	India	-	-	Algae	[25]
Bacillus sp.	PKU-MA00093	×	China	Pacific Ocean	South China Sea	Sponge	[25]

Bacillus sp.	PKU-MA00092	×	-	Pacific Ocean	South China Sea	Sponge	[25]
B. subtilis	B38	×	-	-	-	Unspecified	[25]
Bacillus sp.	WMMC1349	×	United States of America	Atlantic Ocean	Gulf of Mexico	Sponge	[25]
B. subtilis	B5	×	-	Pacific Ocean	-	Sediments	[25]
B. siamensis	-	×	China	Pacific Ocean	South China Sea	Coral	[25]
Bacillus sp.	SCO-147	×	Korea	Pacific Ocean	Sea of Japan	Sediments	[25]
B. spizizenii	-	×	Iraq	Indian Ocean	Persian Gulf	Sediments	[75]
B. spizizenii	-	×	Iraq	Indian Ocean	Persian Gulf	Sediments	[75]
B. spizizenii	-	×	Iraq	Indian Ocean	Persian Gulf	Sediments	[75]
B. spizizenii	-	×	Iraq	Indian Ocean	Persian Gulf	Sediments	[75]
B. spizizenii	-	×	Iraq	Indian Ocean	Persian Gulf	Sediments	[75]
B. spizizenii	-	×	Iraq	Indian Ocean	Persian Gulf	Sediments	[75]
B. spizizenii	-	×	Iraq	Indian Ocean	Persian Gulf	Sediments	[75]
B. spizizenii	-	×	Iraq	Indian Ocean	Persian Gulf	Sediments	[75]
B. spizizenii	-	×	Iraq	Indian Ocean	Persian Gulf	Sediments	[75]
B. spizizenii	-	×	Iraq	Indian Ocean	Persian Gulf	Sediments	[75]
B. spizizenii	-	×	Iraq	Indian Ocean	Persian Gulf	Sediments	[75]
B. spizizenii	-	×	Iraq	Indian Ocean	Persian Gulf	Sediments	[75]
B. spizizenii	-	×	Iraq	Indian Ocean	Persian Gulf	Seawater	[75]
B. spizizenii	-	×	Iraq	Indian Ocean	Persian Gulf	Seawater	[75]
B. spizizenii	-	×	Iraq	Indian Ocean	Persian Gulf	Seawater	[75]
B. spizizenii	-	×	Iraq	Indian Ocean	Persian Gulf	Seawater	[75]
B. spizizenii	-	×	Iraq	Indian Ocean	Persian Gulf	Seawater	[75]
B. spizizenii	-	×	Iraq	Indian Ocean	Persian Gulf	Seawater	[75]
B. spizizenii	-	×	Iraq	Indian Ocean	Persian Gulf	Seawater	[75]
B. spizizenii	-	×	Iraq	Indian Ocean	Persian Gulf	Seawater	[75]

B. haynesii	CD223	✓	Bangladesh	Indian Ocean	Bay of Bengal	Sediments	[76]
Bacillus spp.	-	×	China	Pacific Ocean	South China Sea	Plant	[77]
B. amyloliquefaciens	S13	×	Algeria	Atlantic Ocean	Mediterranean Sea	Algae	[78]
Bacillus sp.	RCS1	✓	China	Pacific Ocean	South China Sea	Fish	[79, 80]
B. cereus	RCS3	✓	China	Pacific Ocean	South China Sea	Fish	[79, 80]
Bacillus sp.	-	×	Saudi Arabia	Indian Ocean	Red Sea	Algae	[81]
B. subtilis	-	✓	China	-	-	Collection	[82]
B. aryabhattai	NM1-A2	×	China	Pacific Ocean	South China Sea	Plant	[83, 84]
B. subtilis	Bsnari	×	Egypt	Indian Ocean	Red Sea	Sediments	[85]
B. velezensis	AG6	×	-	Indian Ocean	Red Sea	Sediments	[86]
B. pumilus	MCB-7	×	India	Indian Ocean	Laccadive Sea	Plant	[87]
B. licheniformis	KB111	×	Thailand	Pacific Ocean	-	Plant	[88]
Bacillus sp.	DL-1	×	-	Ocean	-	Sediments	[88]
B. licheniformis	SWJSS3	×	China	Pacific Ocean	South China Sea	Sediments	[88]
B. subtilis	SMF1	✓	China	-	-	Aquaculture	[89, 90]
B. licheniformis	LMF1	✓	China	-	-	Aquaculture	[89, 90]
B. siamensis	DL3	✓	China	-	-	Aquaculture	[89, 90]
B. taeanensis	SMI-1	×	India	Indian Ocean	Bay of Bengal	Sediments	[91]
B. subtilis	ZK3	×	India	Indian Ocean	Laccadive Sea	Sediments	[92]
B. velezensis	Cpa1-1	✓	China	-	-	Crustacean	[93]
B. licheniformis	4T1C26E	×	India	Indian Ocean	Arabian Sea	Algae	[94]
B. velezensis	5T1C10E	×	India	Indian Ocean	Arabian Sea	Algae	[94]
B. siamensis	LF4	✓	China	-	-	Fish	[95]
B. haynesii	-	×	India	Indian Ocean	Arabian Sea	Sediments	[96]
Bacillus sp.	ZJ318	×	-	Arctic Ocean	-	Sediments	[96]
Bacillus sp.	-	×	Indonesia	-	-	Invertebrate	[96]
B. velezensis	-	×	Saudi Arabia	Indian Ocean	Red Sea	Sediments	[96]
B. subtilis	HR02	×	Italy	Atlantic Ocean	Ligurian Sea	Sediments	[96]

B. cereus	-	✗	Saudi Arabia	Indian Ocean	Red Sea	Sediments	[96]
B. velezensis	FTL	✗	India	Indian Ocean	Laccadive Sea	Sediments	[96]
B. licheniformis	-	✓	China	-	-	Aquaculture	[97]
B. amyloliquefaciens	BTSS3	✓	India	Indian Ocean	Laccadive Sea	Fish	[98]
B. subtilis	-	✗	Malaysia	Indian Ocean	Malacca Strait	Seawater	[99]
B. licheniformis	NCIMB 9375	✗	China	Pacific Ocean	Yellow Sea	Seawater	[99]
Bacillus sp.	APmarine135	✗	Federation of Micronesia	Pacific Ocean	-	Coral	[100]
B. subtilis	W2Z	✓	China	-	-	Aquaculture	[101]
B. aerius	-	✗	Morocco	Atlantic Ocean	Mediterranean Sea	Seawater	[102]
B. velezensis	OQ096500	✗	India	Indian Ocean	Laccadive Sea	Algae	[103]
B. siamensis	OP715893	✗	India	Indian Ocean	Laccadive Sea	Algae	[103]
B. subtilis	OQ0966501	✗	India	Indian Ocean	Laccadive Sea	Algae	[103]
B. filamentous	OP715892	✗	India	Indian Ocean	Laccadive Sea	Algae	[103]
B. siamensis	SK53	✗	India	Indian Ocean	Laccadive Sea	Algae	[103]
B. velezensis	SK54	✗	India	Indian Ocean	Laccadive Sea	Algae	[103]
B. thuringiensis	I.B.6	✗	India	Indian Ocean	Bay of Bengal	Sediments	[104]
B. subtilis	2118	✗	China	Pacific Ocean	East China Sea	Fish	[105]
Bacillus sp.	-	✗	-	-	-	Coral	[106]
Bacillus sp.	-	✗	Germany	Atlantic Ocean	Baltic Sea	Algae	[106]
B. subtilis	KUFA 0162	✗	Thailand	Pacific Ocean	Gulf of Thailand	Sponge	[107]
B. subtilis	KUFA 0163	✗	Thailand	Pacific Ocean	Gulf of Thailand	Sponge	[107]
B. subtilis	KUFA 0165	✗	Thailand	Pacific Ocean	Gulf of Thailand	Sponge	[107]
B. subtilis	KUFA 0169	✗	Thailand	Pacific Ocean	Gulf of Thailand	Sponge	[107]
B. firmus	-	✗	China	Pacific Ocean	South China Sea	Coral	[108]
B. firmus	-	✗	China	Pacific Ocean	South China Sea	Coral	[108]
B. firmus	-	✗	China	Pacific Ocean	South China Sea	Coral	[108]
B. firmus	-	✗	China	Pacific Ocean	South China Sea	Coral	[108]

B. firmus	-	✗	China	Pacific Ocean	South China Sea	Coral	[108]
B. firmus	-	✗	China	Pacific Ocean	South China Sea	Coral	[108]
B. firmus	-	✗	China	Pacific Ocean	South China Sea	Coral	[108]
B. methylotrophicus	B-9987	✗	China	Pacific Ocean	Yellow Sea	Plant	[109]
Bacillus sp.	NIOA284	✗	India	Indian Ocean	Arabian Sea	Algae	[110]
Bacillus spp.	-	✓	Thailand	-	-	Collection	[111]
B. licheniformis	Ba4	✓	China	-	-	Fish	[112]
B. velezensis	T23	✓	China	-	-	Fish	[113, 114]
B. subtilis	MAH84	✗	India	Indian Ocean	Bay of Bengal	Seawater	[115]
B. zhangzhouensis	SK4	✗	India	-	-	Algae	[116]
B. cereus	-	✓	China	Pacific Ocean	Maowei Sea	Plant	[117]
B. cereus	G1-11	✓	China	-	-	Fish	[118-121]
Bacillus sp.	KRF-7	✓	Republic of Korea	-	-	Fish	[122, 123]
Bacillus sp.	PM8313	✓	Republic Of Korea	Pacific Ocean	South Sea of Korea	Fish	[122, 124]
B. subtilis	TISTR-1528	✓	Thailand	-	-	Aquaculture	[125]
B. velezensis	12Y	✗	Taiwan	Pacific Ocean	South China Sea	Sediments	[126]
B. licheniformis	BCLLNF-01	✗	Brazil	Atlantic Ocean	-	Coral	[127]
B. cereus	-	✗	Brazil	Atlantic Ocean	Caribbean Sea	Coral	[127]
B. pumilus	KMM456	✗	Vietnam	Pacific Ocean	South China Sea	Coral	[127]
B. subtilis	NC8	✗	Brazil	Atlantic Ocean	-	Coral	[127]
B. subtilis	MSBN17	✗	India	Indian Ocean	Bay of Bengal	Sponge	[128]
Bacillus sp.	-	✗	Saudi Arabia	Indian Ocean	Red Sea	Seawater	[128]
B. subtilis	AAHM01	✓	Thailand	-	-	Fish	[129]
Bacillus sp.	NCB8	✗	India	Indian Ocean	Bay of Bengal	Plant	[130]
B. velezensis	T20	✓	China	-	-	Fish	[131]
B. velezensis	-	✗	China	-	-	Crustacean	

B. velezensis	PGSAK01	✓	China	Pacific Ocean	South China Sea	Fish	[132]
B. stercoris	PGSAK05	✓	China	Pacific Ocean	South China Sea	Fish	[132]
B. velezensis	PGSAK17	✓	China	Pacific Ocean	South China Sea	Fish	[132]
B. subtilis	PGSAK19	✓	China	Pacific Ocean	South China Sea	Fish	[132]
B. amyloliquefaciens	MS	✗	India	Indian Ocean	Bay of Bengal	Sediments	[133]
B. subtilis	2H11	✗	China	Pacific Ocean	South China Sea	Sediments	[134]
B. subtilis	Sp. 18	✗	China	Pacific Ocean	South China Sea	Sponge	[135]
B. aerius	S-4	✓	India	Indian Ocean	Laccadive Sea	Sediments	[136]
B. altitudinus	S-5	✓	India	Indian Ocean	Laccadive Sea	Sediments	[136]
B. pumilus	G-1	✓	India	Indian Ocean	Laccadive Sea	Fish	[136]
B. licheniformis	FI645	✗	Spain	Atlantic Ocean	Caribbean Sea	Fish	[137, 138]
B. licheniformis	FI658	✗	Spain	Atlantic Ocean	Caribbean Sea	Fish	[137, 138]
B. velezensis	MTCC 13097	✗	India	Indian Ocean	Gulf of Mannar	Algae	[139]
B. pumilus	SE5	✓	China	-	-	Fish	[140, 141]
B. cereus	KMS3-1	✗	India	Indian Ocean	Bay of Bengal	Sediments	[142]
B. cereus	PSMS6	✗	India	Indian Ocean	Bay of Bengal	Sediments	[143]
B. subtilis	JSHY-K3	✓	China	-	-	Aquaculture	[144]
B. subtilis	-	✓	China	-	-	Aquaculture	[145]
B. licheniformis	KDM612	✗	Japan	Pacific Ocean	Suruga Bay	Unspecified	[146]
B. infantis	-	✗	India	Indian Ocean	Arabian Sea	Seawater	[147]
B. mojavensis	A21	✗	Tunisia	Atlantic Ocean	Mediterranean Sea	Seawater	[147]
B. altitudinus	-	✗	India	Indian Ocean	Laccadive Sea	Algae	[147]
B. tequilensis	MTCC13043	✗	India	Indian Ocean	Laccadive Sea	Algae	[148]
B. tequilensis	Bt-RS	✗	China	Pacific Ocean	South China Sea	Plant	[149]
B. licheniformis	B3-15	✗	Italy	Atlantic Ocean	Tyrrhenian Sea	Seawater	[150]
B. altitudinus	FHB1	✗	Philippines	Pacific Ocean	Visayan Sea	Algae	[151]
B. australimaris	FHB2	✗	Philippines	Pacific Ocean	Visayan Sea	Algae	[151]
B. safensis	FHBS	✗	Philippines	Pacific Ocean	Visayan Sea	Algae	[151]

B. subtilis	T4	✗	India	Indian Ocean	Gulf of Mannar	Sediments	[152]
B. pumilus	-	✓	China	Pacific Ocean	Maowei Sea	Plant	[153]
B. subtilis	PSB-1	✗	China	Pacific Ocean	East China Sea	Seawater	[154]
B. paralicheniformis	HR-1	✗	India	Indian Ocean	Arabian Sea	Sediments	[155]
B. haynesii	HR-5	✗	India	Indian Ocean	Arabian Sea	Sediments	[155]
B. velezensis	LSL-68	✗	China	Pacific Ocean	South China Sea	Plant	[156]
B. licheniformis	-	✓	China	-	-	Collection	[157]
B. inaquosorum	BSXE-2102	✓	China	-	-	Aquaculture	[158]
B. vallismortis	VSL-2	✗	India	Indian Ocean	Bay of Bengal	Seawater	[159]
B. subtilis	IS-5	✗	India	Indian Ocean	Bay of Bengal	Seawater	[159]
B. subtilis	EXSP1	✗	Italy	Atlantic Ocean	Adriatic Sea	Seawater	[159]
B. subtilis	PPSL-2	✗	Italy	Atlantic Ocean	Adriatic Sea	Seawater	[159]
B. siamensis	VITAAA3	✗	India	Indian Ocean	Gulf of Mannar	Sediments	[160]
B. velezensis	GXMZU-B1	✗	China	-	-	Aquaculture	[161]
B. velezensis	CM7-4	✗	China	Pacific Ocean	South China Sea	Seawater	[162]
B. inaquosorum	M1	✓	China	Pacific Ocean	Yellow Sea	Sediments	[163]
B. subtilis	BG1	✗	India	Indian Ocean	Laccadive Sea	Plant	[164]
B. subtilis	LC 550	✓	China	-	-	Fish	[165]
B. cereus	PMS1	✗	India	Indian Ocean	Bay of Bengal	Plant	[166]
B. subtilis	PMS8	✗	India	Indian Ocean	Bay of Bengal	Plant	[166]
B. aryabhattai	PYMW	✗	India	Indian Ocean	Bay of Bengal	Plant	[166]
B. cereus	SWB1	✗	India	Indian Ocean	Bay of Bengal	Plant	[166]
B. paramycoides	ST18	✗	India	Indian Ocean	Bay of Bengal	Plant	[166]
B. licheniformis	S-1	✗	China	Pacific Ocean	Yellow Sea	Sediments	[167]
B. cereus	MI1	✗	Brazil	Atlantic Ocean	-	Plant	[168]
B. cereus	MI3	✗	Brazil	Atlantic Ocean	-	Plant	[168]
Bacillus sp.	M4	✗	Brazil	Atlantic Ocean	-	Plant	[168]
B. thuringiensis	MI29	✗	Brazil	Atlantic Ocean	-	Plant	[168]

B. amyloliquefaciens	265XY3	×	India	Indian Ocean	Laccadive Sea	Seawater	[169]
B. subtilis	1245	×	India	Indian Ocean	Arabian Sea	Sponge	[169]
B. infantis	MER_TA_169	×	India	Indian Ocean	Arabian Sea	Algae	[169]
B. amyloliquefaciens	B9	×	India	Indian Ocean	Arabian Sea	Algae	[169]
Bacillus sp.	Z8	×	India	Indian Ocean	Arabian Sea	Algae	[169]
B. velezensis	35	×	India	Indian Ocean	Arabian Sea	Algae	[169]
B. subtilis	A26	×	Tunisia	Atlantic Ocean	Mediterranean Sea	Seawater	[170]
B. subtilis	ULB16	×	India	Indian Ocean	Laccadive Sea	Algae	[170]
B. subtilis	HHEB2	✓	China	-	-	Aquaculture	[171]
B. subtilis	SCSMX-2	×	China	-	-	Fish	[172]
B. velezensis	T-3	×	China	Pacific Ocean	Yellow sea	Invertebrate	[173]
Bacillus sp.	NP5	✓	Indonesia	-	-	Collection	[174]
B. safensis	BS22LVI	×	Republic of Korea	-	-	Crustacean	[175]
B. tropicus	FG2	✓	Taiwan	-	-	Fish	[176]
B. tequilensis	Bt-CO	✓	China	Pacific Ocean	South China Sea	Coral	[177, 178]

Supplementary Table S2- Pathogenic Testing

Strain	Determination of Pathogenicity of *Bacillus* strains														
	Status	HAEM	MORT	SR	VF/ TCG	Other	REF	Strain	Status	HAEM	MORT	SR	VF/ TCG	Other	REF
B. baekryungensis MS1	✓	-	-	↑	-	-	[4]	*B. cereus* RCS3	✓	α	0%	-	-	-	[79, 80]
B. pumilus A97	✓	γ	0%	-	-	-	[5]	B. *subtilis*	✓	-	NC	↑	-	-	[82]
Bacillus sp. MK966345	✓	-	-	↑	-	-	[6]	*B. velezensis* cpa1-1	✓	-	-	↑	-	-	[93]
B. cereus MK966367	✓	-	-	↑	-	-	[6]	*B. subtilis* SMF1	✓	-	0%	-	-	-	[89, 90]
B. licheniformis MK966350	✓	-	-	↑	-	-	[6]	*B. licheniformis* LMF1	✓	-	0%	-	-	-	[89, 90]
B. subtilis MK966347	✓	-	-	↑	-	-	[6]	*B. siamensis* DL3	✓	-	0%	-	-	-	[89, 90]
B. licheniformis ge6-1	✓	γ	-	↑	-	-	[9]	*B. siamensis* LF4	✓	-	0%	NC	-	-	[95]
B. subtilis D5	✓	-	-	↑	-	-	[12]	*B licheniformis*	✓	-	-	-	-	↑health	[97]
B. licheniformis XW15	✓	γ	-	↑	-	-	[17]	*B. amyloliquefaciens* BTSS3	✓	γ	-	-	-	No health effect	[98, 179, 180]
B. subtilis ZF3	✓	γ	-	↑	-	-	[17]	*B. firmus* BSCB-13	✓	-	-	↑	-	-	[52]
B. subtilis DB1	✓	γ	-	↑	-	-	[17]	*B. subtilis* W2Z	✓	γ	-	NC	-	-	[101]
B. cereus PB1-1	✓	γ	-	↑	-	-	[21, 181]	B. aerius	×	β	-	-	-	-	[102]

B. firmus PB106	✓	γ	-	↑	-	-	[21, 181]	*B. siamensis* SK53	✓	γ	-	-	0	-	[103]
B. licheniformis BCR 4-3	✓	γ	-	-	-	-	[27, 182]	*B. velezensis* SK54	✓	γ	-	-	0	-	[103]
B. amyloliquefaciens GB-9	✓	-	-	↑	-	-	[29, 183] [184]	*B. licheniformis* Ba4	✓	γ	0%	NC	0	-	[112]
B. cereus LS2	✓	-	-	↑	-		[30, 31]	*B. velezensis* T23	✓	-	-	NC	-	-	[113, 114]
B. cereus CH	×	β	-	-	143VF	-	[34]	*B. cereus*	✓	-	0%	-	-	↑health	[117]
B. cereus HB	×	β	-	-	140VF	-	[34]	*B. cereus* G1–11	×	-	0%	↑	21VF/TCG	↑health	[118-121]
B. cereus WH	×	β	-	-	141VF	-	[34]	*Bacillus* sp. KRF-7	✓	γ	0%	NC	0	↑health	[123] two study
B. pumilus AQP4275	✓	-	-	-	-	↑ health	[35]	*Bacillus* sp. PM8313	✓	γ	0%	NC	0	↑health	[124] two stdudy
B. toyonensis BCT-7112T	✓	-	-	↑	-	-	[36]	*B. subtilis* TISTR-1528	✓	-	-	-	-	Non-pathogenic	[125]
B. amyloliquefaciens BN06	✓	-	0%	↑	-	-	[37]	*B. subtilis* AAHM01	✓	-	-	NC	-	-	[129]
B. subtilis WN07	✓	-	0%	↑	-	-	[37]	*B. velezensis* T20	✓	-	-	↑	-	-	[131]
B. subtilis MBTDCMFRI Ba37	✓	γ	-	↑	-	↑ to 95% viability	[38]	*B. velezensis* PGSAK01	✓	γ	-	-	0	-	[132]

B. thuringiensis QQ1	✓	γ	0%	↑	-	-	[40, 185]	*B. stercoris* PGSAK05	✓	γ	-	-	0	-	[132]
B. cereus QQ2	✓	γ	0%	↑	-	-	[40, 185]	*B. velezensis* PGSAK17	✓	γ	-	-	0	-	[132]
B. safensis SDG14	✓	γ		-		0 hits in PF	[44]	*B. subtilis* PGSAK19	✓	γ	-	-	1TCG	-	[132]
B. safensis strain SQVG18	✓	-	-	NC	-	-	[47]	*B. licheniformis* FI645	✓	γ	0%	↑	-	-	[137, 138]
B. safensis VQV8	✓	γ	-	-	-	-	[52]	*B. licheniformis* FI658	✓	γ	0%	↑	-	-	[137, 138]
B. velezensis D-18	✓	-	0%	↑	-	↑healt h	[53]	*B. pumilus* SE5	✓	-	0%	NC	-	-	[140, 186]
B. pumilus 36R ATNSAL	~	-	-	-	68VF	-	[56]	*B. subtilis* JSHY-K3	✓	-	-	↑	-	-	[144]
B. safensis 13L LOBSAL	~	-	-	-	57VF	-	[56]	*B. subtilis*	✓	-	-	↑	-	-	[145]
B. velezensis MVCR2	✓	-	-	↑	-	-	[57]	*B. pumilus*	✓	-	NC	NC		-	[153]
Bacillus sp. SW1-1	✓	-	-	↑	-	-	[58]	*B. licheniformis*	✓	-	-	-	-	No health effect	[157]
B. subtilis P2.24.	✓	γ	-	↑	-		[61, 187]	*B. inaquosorum* BSXE-2102	✓	γ	-	↑	50VF 1TCG	-	[158]
B. altitudinus D6.19	✓	γ	-	↑	-		[61, 187]	*B. inaquosorum* M1	✓	γ	0%	↑	-	↑healt h	[163]
B. subtilis FI314	✓	-	100% at 1000u g/mL	↑	-	↑	[62, 188]	*B. subtilis* LC 550	✓	γ	-	↑	-	-	[165]

B. velezensis FI436	✓	-	100% at 1000ug/mL	↑	-	↑	[62, 188]	*B. subtilis* HHEB2	✓	γ	-	-	-	-	[171]
B. pumilus FI464	✓	-	100% at 1000ug/mL	↑	-	↑	[62, 188]	*B. subtilis* SCSMX-2	✓	-	0%	-	-	No significant toxicity	[172]
B. subtilis ABP1	✓	γ	-	↑	-	↓ inflammation	[67, 189]	*B. velezensis* T-3	✓	γ	-	-	-	-	[173]
B. subtilis ABP2	✓	α	-	↑	-	↓ inflammation	[67, 189]	*Bacillus* sp. NP5	✓	-	-	↑	-	-	[174]
B. licheniformis PNB3	✓	-	-	-	0	↑health	[74]	*B. safensis* BS22LVI	✓	-	-	↑	-	-	[175]
B. haynesii CD223	✓	-	0%	↑	0	-	[76]	*B. tropicus* FG2	✓	-	0%	-	-	-	[176, 190]
Bacillus sp. RCS1	✓	γ	0%	-	-	-	[79, 80]	*B. tequilensis* Bt-CO	✓	γ	-	↑	-	↑health	[178]

HAEM: Haemolytic Activity, **MORT:** Mortality, **SR:** Survival Rate, **VF/ TCG:** Virulence Factors/ Toxin Coding Genes, **REF:** References

✓= Criteria Met/ Non-pathogenic, ✗= Criteria Failed/ Pathogenic

↑= Increased Parameter, ↓= Decreased Parameter, **NC**= No change in parameter

Supplementary Table S4- Antibiotic Resistance

	EFSA Guideline for Antimicrobial Drug Resistance										
	Type	Status	VAN	GEN	KAN	STREP	ERY	CLIN	TET	CHLOR	REF
**Standard MIC (mg/L)**	*In vitro*	Cut-off	4	4	8	8	4	4	8	8	[191]
B. pumilus A97	*In vitro*	~	S	S	S	S	S	-	S	S	[5]
B. subtilis KC02	*In vitro*	~	-	-	S	-	-	-	S	-	[7]
B. subtilis KC04	*In vitro*	~	-	-	S	-	-	-	S	-	[7]
B. subtilis KC05	*In vitro*	~	-	-	S	-	-	-	S	-	[7]
B. subtilis KC12	*In vitro*	~	-	-	S	-	-	-	S	-	[7]
B. subtilis KC14	*In vitro*	~	-	-	S	-	-	-	S	-	[7]
B. subtilis KC16	*In vitro*	~	-	-	S	-	-	-	S	-	[7]
B. subtilis KC17	*In vitro*	~	-	-	S	-	-	-	S	-	[7]
B. subtilis KC18	*In vitro*	~	-	-	S	-	-	-	S	-	[7]
B. subtilis KC20	*In vitro*	~	-	-	S	-	-	-	S	-	[7]
B. subtilis KC22	*In vitro*	~	-	-	S	-	-	-	S	-	[7]
B. amyloliquefaciens KC06	*In vitro*	~	-	-	S	-	-	-	S	-	[7]
B. amyloliquefaciens KC08	*In vitro*	~	-	-	S	-	-	-	S	-	[7]
B. amyloliquefaciens KC11	*In vitro*	~	-	-	S	-	-	-	S	-	[7]

B. amyloliquefaciens KC13	*In vitro*	~	-	-	S	-	-	-	S	-	[7]
B. amyloliquefaciens KC15	*In vitro*	~	-	-	S	-	-	-	S	-	[7]
B. licheniformis KC03	*In vitro*	~	-	-	S	-	-	-	S	-	[7]
B. safensis KC10	*In vitro*	~	-	-	S	-	-	-	S	-	[7]
B. pacificus KC07	*In vitro*	~	-	-	S	-	-	-	S	-	[7]
B. pacificus KC09	*In vitro*	~	-	-	S	-	-	-	S	-	[7]
B. halotolerans KC01	*In vitro*	~	-	-	S	-	-	-	S	-	[7]
B. halotolerans KC19	*In vitro*	~	-	-	S	-	-	-	S	-	[7]
B. halotolerans KC21	*In vitro*	~	-	-	S	-	-	-	S	-	[7]
B. licheniformis BCR 4-3	*In vitro*	~	-	S	-	-	-	-	-	-	[27, 182]
B. subtilis MBTDCMFRI Ba37	*In vitro*	~	-	S	S	S	S	-	S	S	[38, 192]
B. thuringiensis QQ1	*In vitro*	~	-	S	-	-	-	-	-	-	[40, 41, 185]
B. cereus QQ2	*In vitro*	~	-	S	-	-	-	-	-	-	[40, 41, 185]
B. safensis SDG14	WGS	✓	A	A	A	A	A	A	A	A	[44]
B. safensis VQV8	*In vitro*	~	S	-	S	R	R	-	S	R	[52]
B. subtilis ABP1	*In vitro*	~	S	S	S	S	S	-	S	S	[67, 189]

B. subtilis ABP2	*In vitro*	~	S	S	S	S	S	-	S	S	[67, 189]
B. licheniformis PNB3	*In vitro*	~	-	S	S	R	-	-	S	-	[74]
Bacillus sp. RCS1	*In vitro*	✓	S	S	S	(Amikacin) S	S	S	(Doxycycline) S	S	[79]
B. cereus RCS3	*In vitro*	~	R	S	S	(Amikacin) S	S	S	(Doxycycline) R	S	[79]
B. aryabhattai NM1-A2	*In vitro*	✓	S	S	-	S	S	-	S	-	[83, 84]
	WGS		A	A	A	A	A	A	A	A	
B licheniformis	*In vitro*	✓	-	S	-	-	S	-	S	S	[95]
	WGS		A	A	A	A	A	A	A	A	
B. amyloliquefaciens BTSS3	*In vitro*	~	S	-	-	-	-	-	S	S	[98, 179, 180]
	WGS		-	-	-	-	-	clbA	tet45	-	
B. siamensis SK53	*In vitro*	~	S	S	-	-	S	S	-	-	[103]
B. velezensis SK54	*In vitro*	~	S	S	-	-	S	S	-	-	[103]
B. licheniformis Ba4	WGS	✓	A	A	A	A	A	A	A	A	[112]
B. cereus G1–11	*In vitro*	✓	S	S	S	Amikacin (S)	S	S	S	S	[118-121]
	WGS		vanRM vanZF	rmtH baeS AAC(2′)-IIb aadA16	rmtH baeS AAC(2′)-IIb aadA16	Erm(50) mgtA	rmtH baeS AAC(2′)-IIb aadA16	Erm(50)	A	A	
Bacillus sp. PM8313	*In vitro*	✓	S	S	S	S	S	S	S	S	[124]

Bacillus sp. KRF-7	*In vitro*	~	-	-	S	S	S	-	S	-	[123]
B. velezensis PGSAK01	*In vitro*	✓	S	S	S	(Amikacin) S	S	S	S	S	[132]
	WGS		A	A	A	A	-	-	tet K	cat	
B. stercoris PGSAK05	*In vitro*	✓	S	S	S	(Amikacin) S	S	S	S	S	[132]
	WGS		A	A	A	A	-	-	A	A	
B. velezensis PGSAK17	*In vitro*	✓	S	S	S	(Amikacin) S	S	S	S	S	[132]
	WGS		A	ant(4')-Ia	ant(4')-Ia	ant(4')-Ia	-	-	tek K	cat	
B. subtilis PGSAK19	*In vitro*	✓	S	S	S	(Amikacin) S	S	S	S	S	[132]
	WGS		A	A	A	A	-	-	A	A	
B. aerius S-4	*In vitro*	~	-	R	R	-	S	S	-	-	[136]
B. altitudinus S-5	*In vitro*	~	-	R	R	-	S	S	-	-	[136]
B. pumilus G-1	*In vitro*	~	-	R	R	-	S	S	-	-	[136]
B. licheniformis FI645	*In vitro*	~	S	S	S	S	R	-	S	R	[137, 138]
B. licheniformis FI658	*In vitro*	~	S	S	S	S	R	-	S	R	[137, 138]
B. inaquosorum BSXE-2102	*In vitro*	✓	S	S	S	S	S	S	S	S	[158]
	WGS		vanT vanW vanY	yykC yykD	yykC yykD	yykC yykD	mphK	Absent	yykC yykD	Absent	
B. inaquosorum M1	*In vitro*	✓	S	S	-	-	S	S	-	-	[163]
	WGS		A	A	A	A	A	A	A	A	
B. subtilis HHEB2	*In vitro*	~	-	S	-	R	S	-	S	-	[171]

B. velezensis T-3	*In vitro*	~	-	-	-	-	S	-	-	S	[173]
B. tequilensis Bt-CO	*In vitro*	~	S	S	S	-	S	-	S	-	[177, 178]

VAN: Vancomycin, **GEN:** Gentamycin, **KAN:** Kanamycin, **STREP:** Streptomycin, **ERY:** Erythromycin, **CLIN:** Clindamycin, **TET:** Tetracycline, **CHLOR:** Chloramphenicol, **REF:** References, **WGS**: Whole Genome Sequencing

✓: Criteria Met, ✗: Criteria Failed, ~: Further Testing Required

S= Susceptible, **R**= Resistant, **A**: Absent, **-**: Not Tested

↑= Increased Parameter, ↓= Decreased Parameter, NC= No change in parameter

Supplementary Table S5- Gut Survivability

Gut Survivability Parameters

***Bacillus* strains**	**STAT**	**CAT**	**pH**	**TEMP (°C)**	**BILE (%)**	**SIJ (SR %)**	**SGJ (SR %)**	**REF**
B. pumilus A97	~	RAN	2-8	-	-	-	-	[5]
		OPT	8	-	-	-	-	
B. subtilis KC02	×	RAN	5-9	30-50	0	-	-	[7]
B. subtilis KC04	×	RAN	5-9	30-50	0	-	-	[7]
B. subtilis KC05	×	RAN	5-9	30-50	0	-	-	[7]
B. subtilis KC12	×	RAN	5-9	30-50	0	-	-	[7]
B. subtilis KC14	×	RAN	5-9	30-50	0	-	-	[7]
B. subtilis KC16	×	RAN	5-9	30-50	0	-	-	[7]
B. subtilis KC17	×	RAN	5-9	30-50	0	-	-	[7]
B. subtilis KC18	×	RAN	5-9	30-50	0	-	-	[7]
B. subtilis KC20	×	RAN	5-9	30-50	0	-	-	[7]
B. subtilis KC22	×	RAN	5-9	30-50	0	-	-	[7]
B. amyloliquefaciens KC06	×	RAN	5-9	30-50	0	-	-	[7]
B. amyloliquefaciens KC08	×	RAN	5-9	30-50	0	-	-	[7]

***Bacillus* strains**	**STAT**	**CAT**	**pH**	**TEMP (°C)**	**BILE (%)**	**SIJ (SR %)**	**SGJ (SR %)**	**REF**
B. velezensis D-18	×	RAN	5-8	-	0-10	-	-	[23, 24]
		OPT	7	-	0	-	-	
B. haynesii CD223	✓	RAN	3-9	-	0.3	-	-	[76]
B. subtilis ABP1	✓	RAN	-	-	-	Survived	Survived	[67, 189]
B. subtilis ABP2	✓	RAN	-	-	-	Survived	Survived	[67, 189]
B. aryabhattai NM1-A2	~	RAN	5.5-8	30-60	-	-	-	[83, 84]
		OPT	6	37	-	-	-	
B. amyloliquefaciens BTSS3	✓	RAN	3-9	8-42	0.3	-	-	[98, 179, 180] [7]
		OPT	-	30-42	-	-	-	
B. siamensis SK53	~	RAN	7-9	-	-	-	-	[103]
		OPT	-	37	-	-	-	
B. velezensis SK54	~	RAN	6-7	-	-	-	-	[103]
		OPT	-	30	-	-	-	[27, 182]
B. cereus G1–11	✓	RAN	3-7	-	0-0.3	-	-	[118]

B. amyloliquefaciens KC11	×	RAN	5-9	30-50	0	-	-	[7]
B. amyloliquefaciens KC13	×	RAN	5-9	30-50	0	-	-	[7]
B. amyloliquefaciens KC15	×	RAN	5-9	30-50	0	-	-	[7]
B. licheniformis KC03	×	RAN	5-9	30-50	0	-	-	[7]
B. safensis KC10	×	RAN	5-9	30-50	0	-	-	[7]
B. pacificus KC07	×	RAN	5-9	30-50	0	-	-	[7]
B. pacificus KC09	×	RAN	5-9	30-50	0	-	-	[7]
B. halotolerans KC01	×	RAN	5-9	30-50	0	-	-	[7]
B. halotolerans KC19	×	RAN	5-9	30-50	0	-	-	[7]
B. halotolerans KC21	×	RAN	5-9	30-50	0	-	-	[7]
B. licheniformis BCR 4-3	~	RAN	6-10	-	-	-	-	[27, 182]
		OPT	6.5	-	-	-	-	
B. subtilis MBTDCMFRI Ba37	~	RAN	4-10	20-55	-	-	-	[38, 192]
	✓	RAN	1.5-9	-	2.5 - 7.5	-	-	
		OPT	7	-	0	-	-	
Bacillus sp. KRF-7	✓	RAN	-	-	0.3	5.96± 0.24	66.85± 4.07	[122, 123]
Bacillus sp. PM8313	✓	RAN	6-8	15-55	0.3	35.11 ±0/16	42.51± 0.19	[122, 124]
		OPT	7	35	-	-	-	
B. velezensis PGSAK01	✓	RAN	1-10	30-80	0.5	-	-	[132]
		OPT	7	37	-	-	-	
B. velezensis PGSAK05	✓	RAN	1-10	30-90	0.5	-	-	[132]
		OPT	7	37	-	-	-	
B. velezensis PGSAK17	✓	RAN	1-10	30-90	0.5	-	-	[132]
		OPT	7	37	-	-	-	
B. velezensis PGSAK19	✓	RAN	1-10	30-90	0.5	-	-	[132]
		OPT	7.5	37	-	-	-	
B. aerius S-4	✓	RAN	3-6.5	30-60	0-10	-	-	[136]
		OPT	6.5	40	0	-	-	

B. thuringiensis QQ1		OPT	7	-	2.5	-	-	[40, 41, 185]
B. cereus QQ2	✓	RAN	1.5-9	-	2.5-7.5	-	-	[40, 41, 185]
		OPT	8	-	2.5	-	-	
B. safensis SDG14	✓	RAN	3-9	-	0.5	-	-	[44]
		OPT	7					
B. safensis strain SQVG18	~	RAN	-	15-55	-	-	-	[47]
		OPT	-	35	-	-	-	
B. safensis VQV8	~	RAN	3-10	25-45	-	-	-	[53]
		OPT	7.5	30	-	-	-	
Bacillus sp. RCS1	✓	RAN	1-9	37-100	0.5	-	-	[79, 80]
		OPT	8	37	-	-	-	
B. cereus RCS3	✓	RAN	1-6	37-100	0.5	-	-	[79, 80]
		OPT	7	37	-	-	-	
B. altitudinus S-5	✓	RAN	3-6.5	30-70	0-10	-	-	[136]
		OPT	6.5	50	0	-	-	
B. pumilus G-1	✓	RAN	3-6.5	30-70	0-10	-	-	[136]
		OPT	6.5	40	0	-	-	
B. inaquosorum BSXE-2102	✓	RAN	7.5	-	-	-	-	[158]
B. inaquosorum M1	~	RAN	3-9	30-90	-	65.67	-	[163]
		OPT	7	40	-	-	-	
B. subtilis HHEB2	~	RAN	5.5-9.5	22-34	-	-	-	[171]
		OPT	7.5	28	-	-	-	
B. velezensis T-3	~	RAN	4-8	20-60	-	-	-	[173]
		OPT	6	30	-	-	-	
B. tequilensis Bt-CO	✓	RAN	2.5-6.8	-	0.15-0.6	94.37	92.59	[177, 178]
		OPT	7.5	-	0.15	-	-	

CAT: Category**, TEMP:** Temperature, **SIJ:** Simulated Intestinal Juice, **SGJ**: Simulated Gastric Juice, **SR:** Survival Rate, **REF:** References, **RAN**: Range, **OPT:** Optimal

✓: Criteria Met, ✗: Criteria Failed, ~: Further Testing Required, **-**: Not Tested

Supplementary Table S5- Gut Colonisation

	Gut Colonisation Parameters							
Bacillus strains	STAT	AGG	HYDRO		CA	GM	WGS	REF
B. pumilus A97	✓	87.88% after 24hrs	Ethyl Acetate	45.05	-	-	-	[5]
			Xylene	46.63				
			Chloroform	45.38				
B. licheniformis BCR 4-3	✓	64.18% at 5hrs	Ethyl Acetate	61.14±0.02	-	-	-	[27, 182]
			p-xylene	62.46±0.05				
			Chloroform	69.63 ± 0.03				
B. safensis SDG14	✓	18.4 ± 0.48 at 3hrs	-	-	45.54± % Hep-2 Cell Line	-	Enolase, Glyceraldehyde-3-phosphate dehydrogenase, Fibronectin/fibrinogen-binding protein, Glycosyl transferases, EpsCD, EpsK, EpsI, Triosephosphate isomerase, FliE-K, FliQR, FliP, RNA polymerase sigma factor, FleN, FlhF, FlhAB, FliLMN, FlbD, FlgDE, FlgBC, FlbB	[44]
Bacillus sp. RCS1	✓	79 ± 0.44 at 24hrs	Ethyl Acetate	97.2%	-	-	-	[79, 80]
			Xylene	89.4%				
			Chloroform	99.1%				
B. cereus RCS3	✓	80.6 ± 0.13 at 24hrs	Ethyl Acetate	97.1%	-	-	-	[79, 80]
			Xylene	92.5%				
			Chloroform	99.1%				
B. velezensis D-18	✓		BSA	<20%	60.33% Intestinal Mucosa			23, 24]
			Polystyrene	<20%				
B. subtilis ABP1	✓	-	-	-	Adherence RTgutGC epithelial cells	-	-	[67, 189]

B. subtilis ABP2	✓	-	-	-	Adherence RTgutGC epithelial cells	-	-	[67, 189]
B. subtilis	~	-	-	-	-	↓Between day 2-8	-	[82]
B. amyloliquefaciens BTSS3	✓	27.22±2.01%	-	32±10.38% after 90mins	Adherence to HEP-2 cell	-	-	[180]
B. cereus G1–11	✓	93.83% at 24hrs	Ethyl Acetate	>90%	-	-	srtA, afuABC, fbpaA, fbpBC, cpaB, rcpC cpaF, tadA lspA, ENO, eno mtnW, gapN, flhAB, flhF, fliC, hag fliD-I, fliMN fliP-S, flgB-E flgG flgKL	[118]
			Xylene	>90%				
			Hexadecane	>90%				
			Hexane	>90%				
Bacillus sp. KRF-7	✓	-	-	-	Spore: 0.55±0.06% Caco-2 cells	-	-	[123]
					Vegetative Cell: 14.77±1.94 % Caco-2 cells			
Bacillus sp. PM8313	✓	-	-	-	61.00 ± 0.05% HT-29 cells	-	-	[124]
B. velezensis PGSAK01	✓	91.45±0.57 at 24hrs	Ethyl Acetate	90.45±3.70	-	-	-	[132]
			Xylene	82.89±1.97	-	-	-	
			Chloroform	95.96±1.66	-	-	-	
B. velezensis PGSAK05	✓	91.64±0.87 at 24hrs	Ethyl Acetate	81.08±1.43	-	-	-	[132]
			Xylene	86.73±2.81	-	-	-	
			Chloroform	94.25±2.49	-	-	-	
B. velezensis PGSAK17	✓	83.47±0.62 at 24hrs	Ethyl Acetate	93.02±2.66	-	-	-	[132]
			Xylene	93.17±2.46	-	-	-	
			Chloroform	93.31±2.68	-	-	-	
	✓		Ethyl Acetate	90.82±3.72	-	-	-	[132]

B. velezensis PGSAK19		88.23±1.05 at 24hrs	Xylene	91.52±2.68	-	-	-	
			Chloroform	87.69±1.74	-	-	-	
B. inaquosorum BSXE-2102	~	-	-	-	-	-	Fibronectin-binding protein, PilA, FlgD, FlgKL, FliE, collagen-binding related protein, EF-Tu, sortase A, FabG, BslAB, EpsA-O	[158]
B. tequilensis Bt-CO	✓	92.59	Xylene	26.36	87,450±641 CFU/g	NC 0-14 days	-	[177, 178]

STAT: Status, **AGG:** Auto-aggregation; **HYDRO**: Hydrophobicity, **CA**: Cell Adhesion, **GM**: Gut Microbiome, **WGS**: Whole Genome Sequencing, **REF:** References, **OPT:** Optimal

✓: Criteria Met, ✖: Criteria Failed, ~: Further Testing Required, **-**: Not Tested

Supplementary Table S6- Enzyme

Strains	Model	CAR	LPS	PRO	CEL	SOD	CAT	OXI	II	TRANS	AKP	ACP	LZM	REF
B. baekryungensis MS1	*In vitro*	-	-	-	-	↑	↑	↑	↑	-	↑	↑	-	[4]
B. pumilus A97	*In vitro*	+	-	+	-	-	-	-	-	-	-	-	-	[5]
	In vivo	-	-	-	-	↓	↓	-	-	-	↓	↓	↑	
Bacillus sp. MK966345	*In vivo*	-	-	-	-	-	-	↑	-	-	-	-	NC	[6]
B. cereus MK966367	*In vivo*	-	-	-	-	-	-	NC	-	-	-	-	↑	[6]
B. licheniformis MK966350	*In vivo*	-	-	-	-	-	-	NC	-	-	-	-	↑	[6]
B. subtilis MK966347	*In vivo*	-	-	-	-	-	-	NC	-	-	-	-	NC	[6]
B. subtilis KC02	*In vitro*	+	-	+	+	-	-	-	-	-	-	-	-	[7]
B. subtilis KC04	*In vitro*	+	-	+	+	-	-	-	-	-	-	-	-	[7]
B. subtilis KC05	*In vitro*	+	-	×	+	-	-	-	-	-	-	-	-	[7]
B. subtilis KC12	*In vitro*	+	-	+	+	-	-	-	-	-	-	-	-	[7]
B. subtilis KC14	*In vitro*	+	-	×	+	-	-	-	-	-	-	-	-	[7]
B. subtilis KC16	*In vitro*	+	-	+	+	-	-	-	-	-	-	-	-	[7]
B. subtilis KC17	*In vitro*	+	-	+	+	-	-	-	-	-	-	-	-	[7]
B. subtilis KC18	*In vitro*	+	-	+	+	-	-	-	-	-	-	-	-	[7]
B. subtilis KC20	*In vitro*	+	-	×	×	-	-	-	-	-	-	-	-	[7]
B. subtilis KC22	*In vitro*	+	-	+	+	-	-	-	-	-	-	-	-	[7]
B. amyloliquefaciens KC06	*In vitro*	+	-	+	+	-	-	-	-	-	-	-	-	[7]

B. amyloliquefaciens KCO8	*In vitro*	+	-	+	+	-	-	-	-	-	-	-	-	[7]
B. amyloliquefaciens KC11	*In vitro*	+	-	+	+	-	-	-	-	-	-	-	-	[7]
B. amyloliquefaciens KC13	*In vitro*	+	-	+	+	-	-	-	-	-	-	-	-	[7]
B. amyloliquefaciens KC15	*In vitro*	+	-	+	+	-	-	-	-	-	-	-	-	[7]
B. licheniformis KC03	*In vitro*	×	-	×	+	-	-	-	-	-	-	-	-	[7]
B. safensis KC10	*In vitro*	×	-	+	×	-	-	-	-	-	-	-	-	[7]
B. pacificus KC07	*In vitro*	+	-	+	+	-	-	-	-	-	-	-	-	[7]
B. pacificus KC09	*In vitro*	+	-	+	+	-	-	-	-	-	-	-	-	[7]
B. halotolerans KC01	*In vitro*	+	-	+	+	-	-	-	-	-	-	-	-	[7]
B. halotolerans KC19	*In vitro*	+	-	+	+	-	-	-	-	-	-	-	-	[7]
B. halotolerans KC21	*In vitro*	+	-	×	+	-	-	-	-	-	-	-	-	[7]
B. licheniformis ge6-1	*In vivo*	↑	↑	↑	↑	↑	↑	-	-	-	-	↑	↑	[9]
B. licheniformis XW15	*In vitro*	-	-	-	-	-	-	-	-	-	-	-	-	[17]
	In vivo	-	-	-	↑	↓	↑	-	-	-	↑	↑	↑	
B. subtilis ZF3	*In vitro*	-	↑	-	-	-	-	-	-	-	-	-	-	[17]
	In vivo	-	-	-	-	↓	↑	-	-	-	↑	↑	↑	

SYNSEA - *B. subtilis*	*In vivo*	-	-	-	-	NC	-	NC	-	-	-	-	↑	[23]
B. licheniformis BCR 4-3	*In vitro*	-	×	×	-	-	×	-	-	-	-	-	-	[27, 182]
B. cereus LS2	*In vivo*	-	-	-	-	↑	↑	-	-	-	↑	↑	-	[30, 31]
B. amyloliquefaciens BN06	*In vivo*	↑	-	↑	-	-	-	-	-	-	-	-	-	[37]
B. subtilis WN07	*In vivo*	↑	-	↑	-	-	-	-	-	-	-	-	-	[37]
B. subtilis MBTDCMFRI Ba37	*In vitro*	+	+	+	+	-	-	-	-	-	-	-	-	[38]
	In vivo	-	-	-	-	↑	↑	-	-	↑	↑	-	-	
B. thuringiensis QQ1	*In vitro*	+	-	+	-	-	-	-	-	-	-	-	-	[40, 41, 185]
	In vivo	↑	↑	↑	-	$\uparrow^{L, S}$	$\uparrow^{L, S}$	$\uparrow^{L, S}$	-	-	×	-	-	
B. cereus QQ2	*In vitro*	+	-	+	-	-	-	-	-	-	↑	-	-	[40, 41, 185]
	In vivo	×	↑	↑	-	$\uparrow^{L, S}$	$\uparrow^{L, S}$	$\uparrow^{L, S}$	↑	-	↑	↑	-	
B. altitudinus 19_A	*In vitro*	-	+	-	+	-	-	-	-	-	-	-	-	[42, 43]
B. safensis SQVG18	*In vivo*	↑	↑	↑	-	↑	-	↑	-	↑	-	-	-	
B. velezensis D-18	*In vitro*	-	-	-	-	-	-	-	-	-	-	-	+	[53]
Bacillus sp. SW1-1	*In vivo*	-	-	-	-	↑	-	↑	-	-	-	-	↑	[57]
B. subtilis S3L1	*In vitro*	+	×	+	×	-	-	-	-	-	-	-	-	[58]
B. subtilis S3L4	*In vitro*	+	+	+	×	-	-	-	-	-	-	-	-	[60]
B. licheniformis S3Z2A	*In vitro*	+	+	×	×	-	-	-	-	-	-	-	-	[60]
B. pumilus S3L6	*In vitro*	+	×	×	×	-	-	-	-	-	-	-	-	[60]

B. halotolerans S1L2	*In vitro*	+	+	×	×	-	-	-	-	-	-	-	-	[60]
B. cereus S1L1	*In vitro*	+	×	×	×	-	-	-	-	-	-	-	-	[60]
B. cereus S3Z2B	*In vitro*	✗	+	×	×	-	-	-	-	-	-	-	-	[60]
B. cereus S3A1	*In vitro*	+	×	×	×	-	-	-	-	-	-	-	-	[60]
B. Licheniformis PNB3	*In vivo*	-	-	-	-	$\uparrow^{HE,S}$	$\uparrow^{HE,S}$	-	-	-	$\uparrow^{HE,S}$	-	$\uparrow^{HE,S}$	[74]
Bacillus sp. RCS1	*In vivo*	↑	↑	↑	↑	$\uparrow^{HK,S}$	$\uparrow^{HK,S}$	$\uparrow^{HK,S}$	-	-	-	-	-	[79]
B. cereus RCS3	*In vivo*	↑	↑	↑	↑	$\uparrow^{HK,S}$	$\uparrow^{HK,S}$	$\uparrow^{HK,S}$	-	-	-	-	-	[79]
B. subtilis	*In vivo*	↑	↑	↑	-	-	-	-	-	-	-	-	-	[82]
B. velezensis cpa1-1	*In vitro*	↑	↑	↑	-	-	-	-	-	-	-	-	-	[93, 193]
B. subtilis SMF1	*In vivo*	NC	$\uparrow^{I}$	$\uparrow^{I}$	-	$\uparrow^{L, S}$	-	-	-	-	NC	$\uparrow^{L}$	$\uparrow^{S}$	[89, 90]
B. licheniformis LMF1	*In vivo*	NC	$\uparrow^{I}$	$\uparrow^{I}$	-	$\uparrow^{L, S}$	-	-	-	-	$\uparrow^{S}$	$\uparrow^{L, S}$	$\uparrow^{L,S}$	[89, 90]
B. siamensis DL3	*In vivo*	NC	$\uparrow^{I}$	$\uparrow^{I}$	-	$\uparrow^{L, S}$	-	-	-	-	$\uparrow^{S}$	$\uparrow^{L, S}$	$\uparrow^{S}$	[89, 90]
B. siamensis LF4	*In vivo*	-	-	-	-	-	-	-	↓	↓	-	-	-	[95, 194]
B. amyloliquefaciens BTSS3	*In vitro*	+	+	+	-	-	+	-	-	-	-	-	-	[98, 179, 180]
B. subtilis W2Z	*In vivo*	↑	NC	↑	-	$\uparrow^{H,HE}$	$\uparrow^{H,HE}$	-	-	-	$\uparrow^{H,HE}$	$\uparrow^{H,HE}$	-	[101]
B. licheniformis Ba4	*In vitro*	✗	+	×	+	-	-	-	-	-	-	-	-	[112]
B. velezensis T23	*In vivo*	↑	↑	↑	-	↑	↑	-	-	-	-	-	-	[113, 114]

B. cereus G1–11	*In vivo*	↑	↓	↑	-	↑	↓	↓	-	-	$\downarrow^{L}\uparrow^{S}$	$\downarrow^{L}$	-	[118-120]
Bacillus sp. KRF-7	*In vivo*	-	-	-	-	↑	-	↑	-	NC	-	-	↑	[123]
Bacillus sp. PM8313	*In vivo*	NC	↑	↑	-	↑	-	NC	-	-	-	-	↑	[124]
B. subtilis TISTR-1528	*In vitro*	-	-	+	-	-	-	-	-	-	-	-	-	[125]
B. subtilis AAHM01	*In vivo*	-	-	-	-	↑	↑	↑	-	-	-	-	↑	[129]
B. velezensis T20	*In vivo*	↑	↑	-	-	-	↑	↑	-	-	↑	↑	-	[131]
B. velezensis PGSAK01	*In vivo*	↑	-	-	-	↑	-	-	-	-	↑	↑	↑	[132]
B. stercoris PGSAK05	*In vivo*	↑	-	-	-	↑	-	-	-	-	↑	↑	↑	[132]
B. velezensis PGSAK17	*In vivo*	↑	-	-	-	↑	-	-	-	-	↑	↑	↑	[132]
B. subtilis PGSAK19	*In vivo*	↑	-	-	-	↑	-	-	-	-	↑	↑	↑	[132]
B. licheniformis FI645	*In vivo*	NC	NC	NC	-	-	-	NC	-	-	-	-	NC	[137, 138]
B. licheniformis FI658	*In vivo*	NC	NC	NC	-	-	-	NC	-	-	-	-	NC	[137, 138]
B. pumilus SE5	*In vivo*	↑	NC	NC	-	↓	-	GSH-Px $\downarrow^{L}$	-	$\uparrow^{S}$	$\uparrow^{S}$	$\uparrow^{S}$	-	[140, 195]
B. subtilis	*In vivo*	-	-	-	-	$\uparrow^{L}$	-	-	-	$\uparrow^{L}$	$\uparrow^{L}$	-	$\uparrow^{L}$	[145]
B. pumilus	*In vivo*	↑	↑	↑	-	$\uparrow^{L}$	$\uparrow^{L}$	GSH-Px $\uparrow^{L}$	-	-	$\uparrow^{S}$	$\uparrow^{S}$	$\uparrow^{S}$	[153]
B. licheniformis	*In vivo*	↓	↓	↓	-	-	-	-	-	-	-	-	-	[157]
	In vitro	+	-	+	-	-	+	-	-	-	-	-	-	[158]

B. inaquosorum BSXE-2102	*In vivo*	-	-	-	-	↑	-	Peroxidase ↑	-	-	$\uparrow^{S}$	$\uparrow^{S}$	$\uparrow^{S}$	
B. inaquosorum M1	*In vivo*	$\uparrow^{I}$	$\uparrow^{I}$	$\uparrow^{I, S}$	-	$\uparrow^{S}$	$\uparrow^{S}$	-	-	-	$\uparrow^{S}$	$\uparrow^{S}$	$\uparrow^{S}$	[163]
B. subtilis LC 550	*In vivo*	NC	↑	↑	-	-	-	-	-	-	-	-	-	[165]
B. tropicus FG2	*In vitro*	+	+	+	+	-	-	-	-	-	-	-	-	[176, 190]
	In vivo	↑	↑	↑	-	↑	-	-	-	-	-	-	$\uparrow^{S}$	
B. tequilensis Bt-CO	*In vitro*	+	+	+	+	-	+	-	-	-	-	-	-	[177, 178]
	In vivo	$\uparrow^{I}$	$\uparrow^{I}$	$\uparrow^{I}$	-	V^{S}	-	$\uparrow^{S}$	-	-	$\uparrow^{S}$	$\uparrow^{S}$	$\uparrow^{S}$	

Enzymes: **CAR**: Carbohydrases; **LPS:** Lipases; **PRO:** Proteases; **CEL:** Cellulase; **SOD:** Superoxide dismutase, **CAT**: Catalase, **OXI:** Other Oxidoreductases; **II:** Innate Immunity; **TRANS:** Transferases; **AKP:** Alkaline Phosphatase; **ACP:** Acid Phosphatase; **LZM**: Lysozyme

^{H}:Haemolymph; ^{HK}: Head Kidney; ^{HP}: Hepatopancreas; ^{I}: Intestine; ^{L}: Liver; ^{S}: Serum; ST: Stomach

+: Activity Present *in vitro*; ×: No activity *in vitro*; ↑:Increased activity *in vivo*; ↓: Decreased activity *in vivo*; NC: No changes; V: Variation between articles;-: Not tested

References

1. W. Sun, W. Wu, X. Liu, D. A. Zaleta-Pinet, and B. R. Clark, "Bioactive compounds isolated from marine-derived microbes in China: 2009-2018," *Marine Drugs,* Review vol. 17, no. 6, 2019, Art no. 339, doi: 10.3390/md17060339.
2. W. Wang *et al.*, "A new thiopeptide antibiotic, micrococcin P3, from a marine-derived strain of the bacterium bacillus stratosphericus," *Molecules,* Article vol. 25, no. 19, 2020, Art no. 4383, doi: 10.3390/molecules25194383.
3. J. A. Nweze *et al.*, "Antibiotics development and the potentials of marine-derived compounds to stem the tide of multidrug-resistant pathogenic bacteria, fungi, and protozoa," *Marine Drugs,* Review vol. 18, no. 3, 2020, Art no. 18030145, doi: 10.3390/md18030145.
4. B. Liu *et al.*, "Bacillus baekryungensis MS1 regulates the growth, non-specific immune parameters and gut microbiota of the sea cucumber Apostichopus japonicus," *Fish & Shellfish Immunology,* vol. 102, pp. 133-139, 2020/07/01/ 2020, doi: https://doi.org/10.1016/j.fsi.2020.04.023.
5. S. Liu *et al.*, "Beneficial effects of a host gut-derived probiotic, Bacillus pumilus, on the growth, non-specific immune response and disease resistance of juvenile golden pompano, Trachinotus ovatus," *Aquaculture,* vol. 514, p. 734446, 2020/01/01/ 2020, doi: https://doi.org/10.1016/j.aquaculture.2019.734446.
6. A. Panigrahi *et al.*, "Bio-augmentation of heterotrophic bacteria in biofloc system improves growth, survival, and immunity of Indian white shrimp Penaeus indicus," *Fish & Shellfish Immunology,* vol. 98, pp. 477-487, 2020/03/01/ 2020, doi: https://doi.org/10.1016/j.fsi.2020.01.021.
7. K. M. Chau, D. Van Quyen, J. M. Fraser, A. T. Smith, T. T. H. Van, and R. J. Moore, "Broad spectrum antimicrobial activities from spore-forming bacteria isolated from the Vietnam Sea," *PeerJ,* Article vol. 8, 2020, Art no. 10117, doi: 10.7717/peerj.10117.
8. J. Cao and B.-G. Wang, "Chemical diversity and biological function of indolediketopiperazines from marine-derived fungi," *Marine Life Science & Technology,* vol. 2, no. 1, pp. 31-40, 2020, doi: 10.1007/s42995-019-00023-0.
9. G. Xiaolong, K. Caihuan, W. Fucun, L. Xian, and L. Ying, "Effects of Bacillus lincheniformis feeding frequency on the growth, digestion and immunity of Haliotis discus hannai," *Fish & Shellfish Immunology,* vol. 96, pp. 1-12, 2020/01/01/ 2020, doi: https://doi.org/10.1016/j.fsi.2019.11.038.
10. U. Mokkala and T. Dethoup, "Effects of co-culturing with live and autoclaved bacillus subtilis on antagonistic activity of marine fungi against plant pathogens," *Journal of Pure and Applied Microbiology,* Article vol. 14, no. 2, pp. 1245-1254, 2020, doi: 10.22207/JPAM.14.2.19.
11. J. Schultz and A. S. Rosado, "Extreme environments: a source of biosurfactants for biotechnological applications," *Extremophiles,* vol. 24, no. 2, pp. 189-206, Mar 2020, doi: 10.1007/s00792-019-01151-2.
12. Y. Ock Kim *et al.*, "Growth promoting activity of Penaeus indicus by secondary metabolite producing probiotic bacterium Bacillus subtilis isolated from the shrimp gut," *Journal of King Saud University - Science,* vol. 32, no. 2, pp. 1641-1646, 2020/03/01/ 2020, doi: https://doi.org/10.1016/j.jksus.2019.12.023.

13. B. F. R. de Oliveira, C. M. Carr, A. D. W. Dobson, and M. S. Laport, "Harnessing the sponge microbiome for industrial biocatalysts," *Appl Microbiol Biotechnol,* vol. 104, no. 19, pp. 8131-8154, Oct 2020, doi: 10.1007/s00253-020-10817-3.
14. Z. Ma, S. Zhang, K. Sun, and J. Hu, "Identification and characterization of a cyclic lipopeptide iturin A from a marine-derived Bacillus velezensis 11-5 as a fungicidal agent to Magnaporthe oryzae in rice," *Journal of Plant Diseases and Protection,* Article vol. 127, no. 1, pp. 15-24, 2020, doi: 10.1007/s41348-019-00282-0.
15. A. Rani *et al.*, "A review on microbial products and their perspective application as antimicrobial agents," *Biomolecules,* Review vol. 11, no. 12, 2021, Art no. 1860, doi: 10.3390/biom11121860.
16. Z. Ma *et al.*, "Isolation and characterization of a new cyclic lipopeptide surfactin from a marine-derived Bacillus velezensis SH-B74," *Journal of Antibiotics,* Article vol. 73, no. 12, pp. 863-867, 2020, doi: 10.1038/s41429-020-0347-9.
17. Z. Feng, X. Song, L. Zhao, and W. Zhu, "Isolation of probiotics and their effects on growth, antioxidant and non-specific immunity of sea cucumber Apostichopus japonicus," *Fish & Shellfish Immunology,* vol. 106, pp. 1087-1094, 2020/11/01/ 2020, doi: https://doi.org/10.1016/j.fsi.2020.08.049.
18. E. N. Kim, M. Gao, H. Choi, and G. S. Jeong, "Marine microorganism-derived macrolactins inhibit inflammatory mediator effects in LPS-induced macrophage and microglial cells by regulating BACH1 and HO-1/Nrf2 signals through inhibition of TLR4 activation," *Molecules,* Article vol. 25, no. 3, 2020, Art no. 656, doi: 10.3390/molecules25030656.
19. J. A. M. de Oliveira, D. E. Williams, R. J. Andersen, M. H. Sarragiotto, and D. C. Baldoqui, "Pumilacidins A-E from sediment-derived bacterium Bacillus sp. 4040 and an their antimicrobial activity evaluation," *Journal of the Brazilian Chemical Society,* Article vol. 31, no. 2, pp. 357-363, 2020, doi: 10.21577/0103-5053.20190188.
20. G. Karanam and M. K. Arumugam, "Reactive oxygen species generation and mitochondrial dysfunction for the initiation of apoptotic cell death in human hepatocellular carcinoma HepG2 cells by a cyclic dipeptide Cyclo(-Pro-Tyr)," *Mol Biol Rep,* vol. 47, no. 5, pp. 3347-3359, May 2020, doi: 10.1007/s11033-020-05407-5.
21. M. del Rocío Vega de la Vega *et al.*, "Reducing stress by improving performance of hatchery-reared Catarina scallop (Argopecten ventricosus) spat with different genera of beneficial microorganisms: A biochemical and molecular analysis," *Aquaculture Reports,* vol. 17, p. 100298, 2020/07/01/ 2020, doi: https://doi.org/10.1016/j.aqrep.2020.100298.
22. V. Gogineni, X. Chen, G. Hanna, D. Mayasari, and M. T. Hamann, "Role of symbiosis in the discovery of novel antibiotics," *J Antibiot (Tokyo),* vol. 73, no. 8, pp. 490-503, Aug 2020, doi: 10.1038/s41429-020-0321-6.
23. L. Monzon-Atienza *et al.*, "Isolation and Characterization of a Bacillus velezensis D-18 Strain, as a Potential Probiotic in European Seabass Aquaculture," *Probiotics Antimicrob Proteins,* vol. 13, no. 5, pp. 1404-1412, Oct 2021, doi: 10.1007/s12602-021-09782-8.
24. L. Monzón-Atienza *et al.*, "Dietary supplementation of Bacillus velezensis improves Vibrio anguillarum clearance in European sea bass by activating essential innate immune mechanisms," *Fish & Shellfish Immunology,* vol. 124, pp. 244-253, 2022/05/01/ 2022, doi: https://doi.org/10.1016/j.fsi.2022.03.032.

25. S. Xiao *et al.*, "Secondary Metabolites from Marine-Derived Bacillus: A Comprehensive Review of Origins, Structures, and Bioactivities," *Mar Drugs,* vol. 20, no. 9, Sep 6 2022, doi: 10.3390/md20090567.
26. S. Zhang, X. Liang, G. M. Gadd, and Q. Zhao, "Marine microbial-derived antibiotics and biosurfactants as potential new agents against catheter-associated urinary tract infections," *Marine Drugs,* Review vol. 19, no. 5, 2021, Art no. 255, doi: 10.3390/md19050255.
27. A. S. Vega-Carranza *et al.*, "Alginate microcapsules as delivery and protective systems of Bacillus licheniformis in a simulated shrimp's digestive tract," *Aquaculture,* vol. 540, p. 736675, 2021/07/15/ 2021, doi: https://doi.org/10.1016/j.aquaculture.2021.736675.
28. H. S. Lee and H. J. Shin, "Anti-mycoplasma activity of bacilotetrins c–e, cyclic lipodepsipeptides from the marine-derived bacillus subtilis and structure revision of bacilotetrins a and b," *Marine Drugs,* Article vol. 19, no. 10, 2021, Art no. 528, doi: 10.3390/md19100528.
29. Y. Li, N. Jiang, W. Zhang, Z. Lv, J. Liu, and H. Shi, "Bacillus amyloliquefaciens-9 reduces somatic cell count and modifies fecal microbiota in lactating goats," *Marine Drugs,* Article vol. 19, no. 8, 2021, Art no. 404, doi: 10.3390/md19080404.
30. Y. Shao, C. Wang, T. Lu, J. Jiang, C. Li, and X. Wang, "Dietary Bacillus cereus LS2 protects juvenile sea cucumber Apostichopus japonicus against Vibrio splendidus infection," *Fish & Shellfish Immunology,* vol. 143, p. 109237, 2023/12/01/ 2023, doi: https://doi.org/10.1016/j.fsi.2023.109237.
31. Z. Hu, W. Zhang, W. Liang, Z. Zhang, M. Guo, and C. Li, "Bacillus cereus LS2 from Apostichopus japonicus antagonizes Vibrio splendidus growth," *Aquaculture,* vol. 531, p. 735983, 2021/01/30/ 2021, doi: https://doi.org/10.1016/j.aquaculture.2020.735983.
32. X. Fu, L. Gong, Y. Liu, Q. Lai, G. Li, and Z. Shao, "Bacillus pumilus Group Comparative Genomics: Toward Pangenome Features, Diversity, and Marine Environmental Adaptation," *Frontiers in Microbiology,* Article vol. 12, 2021, Art no. 571212, doi: 10.3389/fmicb.2021.571212.
33. G. M. Mathew, A. Puthiyamadam, K. Sasikumar, S. Ashoor, and R. K. Sukumaran, "Biological treatment of prawn shell wastes for valorization and waste management," *Bioresource Technology Reports,* vol. 15, p. 100788, 2021/09/01/ 2021, doi: https://doi.org/10.1016/j.biteb.2021.100788.
34. X. Zhao, R. Li, H. Dang, L. Wang, S. Fu, and J. Ding, "Comparison of whole genome sequences of three Bacillus cereus strains reveals the food safety risks of Apostichopus japonicus in China," *Aquaculture Reports,* vol. 20, p. 100649, 2021/07/01/ 2021, doi: https://doi.org/10.1016/j.aqrep.2021.100649.
35. E. Bonos *et al.*, "Effect of Bacillus pumilus supplementation on performance, intestinal morphology, gut microflora and meat quality of broilers fed different energy concentrations," *Animal Feed Science and Technology,* vol. 274, p. 114859, 2021/04/01/ 2021, doi: https://doi.org/10.1016/j.anifeedsci.2021.114859.
36. M. A. Liñan-Vidriales *et al.*, "Effect of rice bran fermented with Bacillus and Lysinibacillus species on dynamic microbial activity of Pacific white shrimp (Penaeus vannamei)," *Aquaculture,* vol. 531, p. 735958, 2021/01/30/ 2021, doi: https://doi.org/10.1016/j.aquaculture.2020.735958.

37. K. Saravanan *et al.*, "Effects of single and multi-strain probiotics on the growth, hemato-immunological, enzymatic activity, gut morphology and disease resistance in Rohu, Labeo rohita," *Aquaculture,* vol. 540, p. 736749, 2021/07/15/ 2021, doi: https://doi.org/10.1016/j.aquaculture.2021.736749.
38. A. V. Nair, M. Leo Antony, N. K. Praveen, P. Sayooj, T. Raja Swaminathan, and K. K. Vijayan, "Evaluation of in vitro and in vivo potential of Bacillus subtilis MBTDCMFRI Ba37 as a candidate probiont in fish health management," *Microbial Pathogenesis,* vol. 152, p. 104610, 2021/03/01/ 2021, doi: https://doi.org/10.1016/j.micpath.2020.104610.
39. R. Singh, N. Chauhan, and M. Kuddus, "Exploring the therapeutic potential of marine-derived bioactive compounds against COVID-19," *Environ Sci Pollut Res Int,* vol. 28, no. 38, pp. 52798-52809, Oct 2021, doi: 10.1007/s11356-021-16104-6.
40. R. Ghanei-Motlagh *et al.*, "Quorum quenching probiotics modulated digestive enzymes activity, growth performance, gut microflora, haemato-biochemical parameters and resistance against Vibrio harveyi in Asian seabass (Lates calcarifer)," *Aquaculture,* vol. 531, p. 735874, 2021/01/30/ 2021, doi: https://doi.org/10.1016/j.aquaculture.2020.735874.
41. R. Ghanei-Motlagh *et al.*, "Feed supplementation with quorum quenching probiotics with anti-virulence potential improved innate immune responses, antioxidant capacity and disease resistance in Asian seabass (Lates calcarifer)," *Aquaculture,* vol. 535, p. 736345, 2021/03/30/ 2021, doi: https://doi.org/10.1016/j.aquaculture.2021.736345.
42. N. B. Comba-González, D. Chaves-Moreno, J. Santamaría-Vanegas, and D. Montoya-Castaño, "A pan-genomic assessment: Delving into the genome of the marine epiphyte Bacillus altitudinis strain 19_A and other very close Bacillus strains from multiple environments," *Heliyon,* vol. 10, no. 7, p. e27820, 2024/04/15/ 2024, doi: https://doi.org/10.1016/j.heliyon.2024.e27820.
43. N. B. Comba Gonzalez, D. Montoya Castano, and J. S. Montana Lara, "Genome sequence of the epiphytic bacteria Bacillus altitudinis strain 19_A, isolated from the marine macroalga Ulva lactuca," *Biotechnol Rep (Amst),* vol. 30, p. e00634, Jun 2021, doi: 10.1016/j.btre.2021.e00634.
44. B. E. Saidumohamed and S. Ganapathy Bhat, "Indian oil sardine (Sardinella longiceps) gut derived Bacillus safensis SDG14 with enhanced probiotic competence for food and feed applications," *Food Research International,* vol. 150, p. 110475, 2021/12/01/ 2021, doi: https://doi.org/10.1016/j.foodres.2021.110475.
45. N. Barzkar *et al.*, "Marine Bacterial Esterases: Emerging Biocatalysts for Industrial Applications," *Appl Biochem Biotechnol,* vol. 193, no. 4, pp. 1187-1214, Apr 2021, doi: 10.1007/s12010-020-03483-8.
46. C. Gao *et al.*, "New 24-Membered Macrolactins Isolated from Marine Bacteria Bacillus siamensis as Potent Fungal Inhibitors against Sugarcane Smut," *Journal of Agricultural and Food Chemistry,* Article vol. 69, no. 15, pp. 4392-4401, 2021, doi: 10.1021/acs.jafc.0c07415.
47. M. Zhang *et al.*, "Study of fermented feed by mixed strains and their effects on the survival, growth, digestive enzyme activity and intestinal flora of Penaeus vannamei," *Aquaculture,* vol. 530, p. 735703, 2021/01/15/ 2021, doi: https://doi.org/10.1016/j.aquaculture.2020.735703.

48. G. Altuğ, P. S. Çiftçi Türetken, S. Kalkan, and B. Topaloğlu, "The Distribution and Antibacterial Activity of Marine Sponge-Associated Bacteria in the Aegean Sea and the Sea of Marmara, Turkey," *Current Microbiology,* Article vol. 78, no. 6, pp. 2275-2290, 2021, doi: 10.1007/s00284-021-02489-7.
49. L. Li *et al.*, "The inhibitory effect of Bacillus amyloliquefaciens L1 on Aeromonas hydrophila and its mechanism," *Aquaculture,* vol. 539, p. 736590, 2021/06/30/ 2021, doi: https://doi.org/10.1016/j.aquaculture.2021.736590.
50. F. Feliatra, U. M. Batubara, Y. Nurulita, I. Lukistyowati, and J. Setiaji, "The potentials of secondary metabolites from Bacillus cereus SN7 and Vagococcus fluvialis CT21 against fish pathogenic bacteria," *Microbial Pathogenesis,* vol. 158, p. 105062, 2021/09/01/ 2021, doi: https://doi.org/10.1016/j.micpath.2021.105062.
51. J. Guo, W. Wang, H. Zhao, Y. Luo, M. Wan, and Y. Li, "A new PMA-qPCR method for rapid and accurate detection of viable bacteria and spores of marine-derived Bacillus velezensis B-9987," *Journal of Microbiological Methods,* Article vol. 199, 2022, Art no. 106537, doi: 10.1016/j.mimet.2022.106537.
52. P. Kolanchinathan, P. R. Kumari, K. Raja, G. John, and A. Balasundaram, "Analysis of feed composition and growth parameters of Penaeus monodon supplemented with two probiotic species and formulated diet," *Aquaculture,* vol. 549, p. 737740, 2022/02/25/ 2022, doi: https://doi.org/10.1016/j.aquaculture.2021.737740.
53. V. Huynh-Phuoc *et al.*, "Bacillus safensis isolated from white-leg shrimp, Penaeus vannamei in Taiwan with antagonistic activity against common Vibrio pathogens," *Biocatalysis and Agricultural Biotechnology,* vol. 44, p. 102477, 2022/09/01/ 2022, doi: https://doi.org/10.1016/j.bcab.2022.102477.
54. W. Ren *et al.*, "Cross-habitat distribution pattern of Bacillus communities and their capacities of producing industrial hydrolytic enzymes in Paracel Islands: Habitat-dependent differential contributions of the environment," *Journal of Environmental Management,* vol. 323, p. 116252, 2022/12/01/ 2022, doi: https://doi.org/10.1016/j.jenvman.2022.116252.
55. J.-W. Lee *et al.*, "Dietary SYNSEA probiotic improves the growth of white shrimp, Litopenaeus vannamei and reduces the risk of Vibrio infection via improving immunity and intestinal microbiota of shrimp," *Fish & Shellfish Immunology,* vol. 127, pp. 482-491, 2022/08/01/ 2022, doi: https://doi.org/10.1016/j.fsi.2022.06.071.
56. R. Sánchez-Díaz *et al.*, "Draft genome sequences of Bacillus pumilus 36R ATNSAL and B. safensis 13L LOBSAL, two potential candidate probiotic strains for shrimp aquaculture," *Journal of Global Antimicrobial Resistance,* vol. 31, pp. 304-308, 2022/12/01/ 2022, doi: https://doi.org/10.1016/j.jgar.2022.10.002.
57. Y. Dang *et al.*, "Effects of probiotics on growth, the toll-like receptor mediated immune response and susceptibility to Aeromonas salmonicida infection in rainbow trout Oncorhynchus mykiss," *Aquaculture,* vol. 561, p. 738668, 2022/12/15/ 2022, doi: https://doi.org/10.1016/j.aquaculture.2022.738668.
58. M.-G. Kim *et al.*, "Evaluation of Bacillus sp. SW1-1 as a dietary additive in diets for olive flounder Paralichthys olivaceus," *Animal Feed Science and Technology,* vol. 290, p. 115367, 2022/08/01/ 2022, doi: https://doi.org/10.1016/j.anifeedsci.2022.115367.

59. C.-M. Hung *et al.*, "Exposure of Goniopora columna to polyethylene microplastics (PE-MPs): Effects of PE-MP concentration on extracellular polymeric substances and microbial community," *Chemosphere,* vol. 297, p. 134113, 2022/06/01/ 2022, doi: https://doi.org/10.1016/j.chemosphere.2022.134113.
60. L. Priscilla, M. Rajeev, S. K. Pandian, and M. E, "Gut associated culturable bacterial community in intertidal polychaete worms (Annelida: Polychaeta), their characterization and implications in captive shrimp aquaculture," *Regional Studies in Marine Science,* vol. 52, p. 102274, 2022/05/01/ 2022, doi: https://doi.org/10.1016/j.rsma.2022.102274.
61. D. Aribah, Widanarni, and A. T. Wahyudi, "The effectiveness of marine bacterial microcapsules in controlling vibriosis disease caused by the infection of Vibrio parahaemolyticus in white shrimp Litopenaeus vannamei," *Aquaculture,* vol. 549, 2022, doi: 10.1016/j.aquaculture.2021.737795.
62. R. A. Santos *et al.*, "In vitro modulation of gilthead seabream (Sparus aurata L.) leukocytes by Bacillus spp. extracellular molecules upon bacterial challenge," *Fish & Shellfish Immunology,* vol. 121, pp. 285-294, 2022/02/01/ 2022, doi: https://doi.org/10.1016/j.fsi.2022.01.002.
63. R. Murugan, A. Guru, B. Haridevamuthu, G. Sudhakaran, A. Arshad, and J. Arockiaraj, "Lantibiotics: an antimicrobial asset in combating aquaculture diseases," *Aquaculture International,* vol. 30, no. 5, pp. 2365-2387, 2022, doi: 10.1007/s10499-022-00908-5.
64. A. L. Khan *et al.*, "Mangrove's rhizospheric engineering with bacterial inoculation improve degradation of diesel contamination," *Journal of Hazardous Materials,* vol. 423, p. 127046, 2022/02/05/ 2022, doi: https://doi.org/10.1016/j.jhazmat.2021.127046.
65. P. Y. Qian, A. Cheng, R. Wang, and R. Zhang, "Marine biofilms: diversity, interactions and biofouling," *Nat Rev Microbiol,* vol. 20, no. 11, pp. 671-684, Nov 2022, doi: 10.1038/s41579-022-00744-7.
66. B. Balaji-Prasath *et al.*, "Methods to control harmful algal blooms: a review," *Environmental Chemistry Letters,* vol. 20, no. 5, pp. 3133-3152, 2022, doi: 10.1007/s10311-022-01457-2.
67. F. Docando *et al.*, "Mucosal and systemic immune effects of Bacillus subtilis in rainbow trout (Oncorhynchus mykiss)," *Fish & Shellfish Immunology,* vol. 124, pp. 142-155, 2022/05/01/ 2022, doi: https://doi.org/10.1016/j.fsi.2022.03.040.
68. S. Vicente-Gil *et al.*, "Bacillus subtilis supplemented feeding as a method to increase IgM titers and affinity in response to fish vaccination," *Fish & Shellfish Immunology,* vol. 162, p. 110335, 2025/07/01/ 2025, doi: https://doi.org/10.1016/j.fsi.2025.110335.
69. Y. Dong, G.-L. Song, and D. Zheng, "Naturally effective inhibition of microbial corrosion by bacterium-alga symbiosis on 304 stainless steel," *Journal of Cleaner Production,* vol. 356, p. 131823, 2022/07/01/ 2022, doi: https://doi.org/10.1016/j.jclepro.2022.131823.
70. C. V. Anh, J. S. Kang, H. S. Lee, P. T. H. Trinh, C. S. Heo, and H. J. Shin, "New Glycosylated Secondary Metabolites from Marine-Derived Bacteria," *Marine Drugs,* Article vol. 20, no. 7, 2022, Art no. 464, doi: 10.3390/md20070464.
71. L. Qin, K. Yong, X. Y. Lian, and Z. Zhang, "New metabolites (±)-bacillipyrrole A and bacillipyrazine A from the Mariana Trench-associated bacterium Bacillus subtilis SY2101," *Phytochemistry Letters,* Article vol. 49, pp. 79-82, 2022, doi: 10.1016/j.phytol.2022.03.010.

72. I. A. Silva *et al.*, "Oil spills: impacts and perspectives of treatment technologies with focus on the use of green surfactants," *Environ Monit Assess,* vol. 194, no. 3, p. 143, Feb 4 2022, doi: 10.1007/s10661-022-09813-z.
73. K. Chakraborty, V. K. Kizhakkekalam, and M. Joy, "Polyketide-derived macrobrevins from marine macroalga-associated Bacillus amyloliquefaciens as promising antibacterial agents against pathogens causing nosocomial infections," *Phytochemistry,* vol. 193, p. 112983, 2022/01/01/ 2022, doi: https://doi.org/10.1016/j.phytochem.2021.112983.
74. H. Cao, X. Huang, Y. Gu, X. Zheng, L. Xu, and C. Gai, "Protective effects of Bacillus licheniformis against Citrobacter freundii infection in Chinese mitten crab Eriocheir sinensis," *Journal of Invertebrate Pathology,* vol. 193, p. 107805, 2022/09/01/ 2022, doi: https://doi.org/10.1016/j.jip.2022.107805.
75. E. A. Al-Imara, A. A. R. Jassim, L. J. Mohammed, S. M. Al-Shatty, and L. Abdulazeem, "Secondary metabolites of marine-derived Bacillus spizizenii against the enteric redmouth disease in common carp, Cyprinus carpio," *International Journal of Aquatic Biology,* Article vol. 10, no. 2, pp. 102-110, 2022, doi: 10.22034/ijab.v10i2.1582.
76. M. M. Rahman *et al.*, "Suppression of Streptococcosis and Modulation of the Gut Bacteriome in Nile Tilapia (Oreochromis niloticus) by the Marine Sediment Bacteria Bacillus haynesii and Advenella mimigardefordensis," *Microbiology Spectrum,* vol. 10, no. 6, 2022/11/21/ 2022, doi: https://doi.org/10.1128/spectrum.02542-22.
77. G.-H. Liu *et al.*, "Temperature drives the assembly of Bacillus community in mangrove ecosystem," *Science of The Total Environment,* vol. 846, p. 157496, 2022/11/10/ 2022, doi: https://doi.org/10.1016/j.scitotenv.2022.157496.
78. C. Cardoso, C. Afonso, R. Quintã, and N. M. Bandarra, "The brown seaweed genus Zonaria: major features, biotechnological potential, and applications," *Journal of Applied Phycology,* vol. 34, no. 5, pp. 2243-2257, 2022, doi: 10.1007/s10811-022-02773-4.
79. E. Amenyogbe, J.-s. Huang, G. Chen, and W.-z. Wang, "Probiotic Potential of Indigenous (Bacillus sp. RCS1, Pantoea agglomerans RCS2, and Bacillus cereus strain RCS3) Isolated From Cobia Fish (Rachycentron canadum) and Their Antagonistic Effects on the Growth of Pathogenic Vibrio alginolyticus, Vibrio harveyi, Streptococcus iniae, and Streptococcus agalactiae," *Frontiers in Marine Science,* vol. 8, 2021, doi: 10.3389/fmars.2021.672213.
80. E. Amenyogbe, J.-d. Zhang, J.-s. Huang, and G. Chen, "The efficiency of indigenous isolates Bacillus sp. RCS1 and Bacillus cereus RCS3 on growth performance, blood biochemical indices and resistance against Vibrio harveyi in cobia fish (Rachycentron canadum) juveniles," *Aquaculture Reports,* vol. 25, p. 101241, 2022/08/01/ 2022, doi: https://doi.org/10.1016/j.aqrep.2022.101241.
81. N. M. S. Moubayed, H. J. Al Houri, and S. I. Bukhari, "Turbinaria ornata and its associated epiphytic Bacillus sp. A promising molecule supplier to discover new natural product approaches," *Saudi J Biol Sci,* vol. 29, no. 4, pp. 2532-2540, Apr 2022, doi: 10.1016/j.sjbs.2021.12.041.
82. J. Sahandi, P. Sorgeloos, K. W. Tang, F. Mu, T. Mayor, and W. Zhang, "Beneficial microbes to suppress Vibrio and improve the culture performance of copepod Tigriopus japonicus Mori," *Microbial Pathogenesis,* vol. 183, p. 106334, 2023/10/01/ 2023, doi: https://doi.org/10.1016/j.micpath.2023.106334.

83. H. Sun *et al.*, "Discovery and interaction of four key biosynthetic enzymes under co-regulation for dopamine biosynthesis with marine Meyerozyma guilliermondii GXDK6 and Bacillus aryabhattai NM1-A2," *Int J Biol Macromol,* vol. 309, no. Pt 2, p. 142821, May 2025, doi: 10.1016/j.ijbiomac.2025.142821.
84. M. Kashif *et al.*, "Deciphering the biodesulfurization pathway employing marine mangrove Bacillus aryabhattai strain NM1-A2 according to whole genome sequencing and transcriptome analyses," *Genomics,* vol. 115, no. 3, p. 110635, May 2023, doi: 10.1016/j.ygeno.2023.110635.
85. M. S. Selim, M. M. Abo Elsoud, M. N. M. E. Sanad, N. A. Elattal, H. M. Rifaat, and S. S. Mohamed, "Enzymatic debittering of citrus juices: Optimization, modeling, and characterization of naringinase production from marine Bacillus subtilis strain BSnari," *Biocatalysis and Agricultural Biotechnology,* vol. 53, 2023, doi: 10.1016/j.bcab.2023.102853.
86. M. A. Alharbi *et al.*, "In vitro studies on the pharmacological potential, anti-tumor, antimicrobial, and acetylcholinesterase inhibitory activity of marine-derived Bacillus velezensis AG6 exopolysaccharide," *RSC Advances,* Article vol. 13, no. 38, pp. 26406-26417, 2023, doi: 10.1039/d3ra04009g.
87. D. Thakur, A. Chauhan, P. Jhilta, R. Kaushal, and B. Dipta, "Microbial chitinases and their relevance in various industries," *Folia Microbiol (Praha),* vol. 68, no. 1, pp. 29-53, Feb 2023, doi: 10.1007/s12223-022-00999-w.
88. H. Yao *et al.*, "Microbial-derived salt-tolerant proteases and their applications in high-salt traditional soybean fermented foods: a review," *Bioresour Bioprocess,* vol. 10, no. 1, p. 82, Nov 18 2023, doi: 10.1186/s40643-023-00704-w.
89. S. Ma, D. Yu, Q. Liu, M. Zhao, C. Xu, and J. Yu, "Relationship between immune performance and the dominant intestinal microflora of turbot fed with different Bacillus species," *Aquaculture,* vol. 549, p. 737625, 2022/02/25/ 2022, doi: https://doi.org/10.1016/j.aquaculture.2021.737625.
90. Y. Ye *et al.*, "Nutritional composition of fresh carcass of turbot fed with Bacillus subtilis SMF1, B. licheniformis LMF1 and B. siamensis DL3 and its relationship with intestinal flora," *Animal Feed Science and Technology,* vol. 300, p. 115627, 2023/06/01/ 2023, doi: https://doi.org/10.1016/j.anifeedsci.2023.115627.
91. M. Sarvepalli, A. Velidandi, and N. Korrapati, "Optimization of Siderophore Production in Three Marine Bacterial Isolates along with Their Heavy-Metal Chelation and Seed Germination Potential Determination," *Microorganisms,* Article vol. 11, no. 12, 2023, Art no. 2873, doi: 10.3390/microorganisms11122873.
92. S. S. Shettar, Z. K. Bagewadi, H. N. Kolvekar, T. M. Yunus Khan, and S. M. Shamsudeen, "Optimization of subtilisin production from Bacillus subtilis strain ZK3 and biological and molecular characterization of synthesized subtilisin capped nanoparticles," *Saudi Journal of Biological Sciences,* vol. 30, no. 11, p. 103807, 2023/11/01/ 2023, doi: https://doi.org/10.1016/j.sjbs.2023.103807.
93. Z. Jiang *et al.*, "Pathogenicity of Aeromonas salmonicida and protection effect of Bacillus velezensis on Macrobrachium nipponense against A. salmonicida," *Aquaculture Reports,* vol. 31, p. 101677, 2023/08/01/ 2023, doi: https://doi.org/10.1016/j.aqrep.2023.101677.
94. D. E. Mathew *et al.*, "Performance evaluation and yield optimization of L-glutaminase free L-asparaginase from seaweed-associated bacteria," *Bioresource Technology Reports,* vol. 23, 2023, doi: 10.1016/j.biteb.2023.101534.

95. Z.-Y. Liu *et al.*, "Probiotic components of Bacillus siamensis LF4 mitigated β-conglycinin caused cell injury via modulating TLR2/MAPKs/NF-κB signaling in Lateolabrax maculatus," *Fish & Shellfish Immunology,* vol. 141, p. 109010, 2023/10/01/ 2023, doi: https://doi.org/10.1016/j.fsi.2023.109010.
96. H. Yao *et al.*, "Screening of marine sediment-derived microorganisms and their bioactive metabolites: a review," *World J Microbiol Biotechnol,* vol. 39, no. 7, p. 172, Apr 28 2023, doi: 10.1007/s11274-023-03621-4.
97. L. Jiao, X. Feng, S. Jin, J. Xie, X. Guo, and R. Ma, "Transcriptome analysis of Cryptocaryon irritans tomont responding to Bacillus licheniformis treatment," *Fish & Shellfish Immunology,* vol. 140, p. 108943, 2023/09/01/ 2023, doi: https://doi.org/10.1016/j.fsi.2023.108943.
98. V. D'Rose and S. G. Bhat, "Whole genome sequence analysis enabled affirmation of the probiotic potential of marine sporulater Bacillus amyloliquefaciens BTSS3 isolated from Centroscyllium fabricii," *Gene,* vol. 864, p. 147305, 2023/05/15/ 2023, doi: https://doi.org/10.1016/j.gene.2023.147305.
99. B. Murtaza, M. S. Rahman, C. C. Xu, T. Zhu, and W. Qin, "Environmental Impact Associated with Oil and Grease and Their Emerging Mitigation Strategies," *Waste and Biomass Valorization,* vol. 15, no. 7, pp. 3913-3928, 2024, doi: 10.1007/s12649-024-02425-3.
100. H. Y. Kim *et al.*, "2,4,6-Triphenyl-1-hexene, an Anti-Melanogenic Compound from Marine-Derived Bacillus sp. APmarine135," *Marine Drugs,* Article vol. 22, no. 2, 2024, Art no. 72, doi: 10.3390/md22020072.
101. Z.-L. Zhang *et al.*, "A newly isolated strain of Bacillus subtilis W2Z exhibited probiotic effects on juvenile red claw crayfish, Cherax quadricarinatus," *Aquaculture,* vol. 585, p. 740700, 2024/05/15/ 2024, doi: https://doi.org/10.1016/j.aquaculture.2024.740700.
102. F. Kadiri, A. Ezaouine, M. Blaghen, F. Bennis, and F. Chegdani, "Antibiofilm potential of biosurfactant produced by Bacillus aerius against pathogen bacteria," *Biocatalysis and Agricultural Biotechnology,* vol. 56, p. 102995, 2024/02/01/ 2024, doi: https://doi.org/10.1016/j.bcab.2023.102995.
103. K. Shanoona, K. Chakraborty, C. Varghese, and R. D. Chakraborty, "Antibiotic properties of seaweed-associated heterotrophic Bacilli against drug-resistant pathogens," *Gene Reports,* vol. 37, 2024, doi: 10.1016/j.genrep.2024.102022.
104. M. Karuppusamy, S. S. Kumar, H. Selvam, K. Sangapillai, K. K. Kamachisundaram, and B. K. Rama, "Anti-quorum Sensing Activity and Bioactive Components of Marine-derived Bacteria," *Journal of Pure and Applied Microbiology,* Article vol. 18, no. 3, pp. 2047-2056, 2024, doi: 10.22207/JPAM.18.3.55.
105. C. Yan *et al.*, "Bacillus subtilis 2118 exhibits bactericidal activity due to an inserted fish cDNA library," *Aquaculture,* vol. 593, p. 741300, 2024/12/15/ 2024, doi: https://doi.org/10.1016/j.aquaculture.2024.741300.
106. P. V. Bhagwat, C. Ravindran, and L. Irudayarajan, "Beneficial properties of mucus in coral adaptations and ecological interactions," *Marine Biology,* vol. 171, no. 2, 2024, doi: 10.1007/s00227-023-04372-4.
107. Y. Nujthet, C. Kaewkrajay, A. Kijjoa, and T. Dethoup, "Biocontrol efficacy of antagonists Trichoderma and Bacillus against post-harvest diseases in mangos," *European Journal of Plant Pathology,* Article vol. 168, no. 2, pp. 315-327, 2024, doi: 10.1007/s10658-023-02757-1.

108. X. Liao *et al.*, "Biodiversity and antifouling activity of microbes associated with gorgonian corals Leptogorgia rigida and Menella kanisa from the South China Sea," *Mar Environ Res,* vol. 202, p. 106783, Nov 2024, doi: 10.1016/j.marenvres.2024.106783.
109. S. Xu, Z. Liu, P. Ren, Y. Liu, F. Xiao, and W. Li, "BmfR, a novel GntR family regulator, regulates biofilm formation in marine-derived, Bacillus methylotrophicus B-9987," *Microbiological Research,* Article vol. 287, 2024, Art no. 127859, doi: 10.1016/j.micres.2024.127859.
110. A. S. Jagtap, C. S. Manohar, and N. S. Kadam, "Characterization of bioactive porphyran oligosaccharides produced using porphyranase from Bacillus sp. NIOA284," *Process Biochemistry,* vol. 137, pp. 187-196, 2024/02/01/ 2024, doi: https://doi.org/10.1016/j.procbio.2024.01.007.
111. J. Pekkoh *et al.*, "Co-bioaugmentation with microalgae and probiotic bacteria: Sustainable solutions for upcycling of aquaculture wastewater and agricultural residues into microbial-rice bran complexes," *Environmental Research,* vol. 261, p. 119760, 2024/11/15/ 2024, doi: https://doi.org/10.1016/j.envres.2024.119760.
112. Y. Jin, C. Chen, C. Ai, W. Song, and J. Zhao, "Development of probiotic complex based on the synthetic microbial community and probiotic effects in farming Yunlong grouper," *Aquaculture,* vol. 586, p. 740708, 2024/05/30/ 2024, doi: https://doi.org/10.1016/j.aquaculture.2024.740708.
113. F. Zhang *et al.*, "Nuclease treatment enhances the probiotic effect of Bacillus velezensis T23 on hepatic steatosis and inflammation induced by high-fat diet in zebrafish," *Aquaculture,* vol. 562, 2023, doi: 10.1016/j.aquaculture.2022.738801.
114. H. Yang *et al.*, "Dietary Bacillus velezensis T23 fermented products supplementation improves growth, hepatopancreas and intestine health of Litopenaeus vannamei," *Fish & Shellfish Immunology,* vol. 149, p. 109595, 2024/06/01/ 2024, doi: https://doi.org/10.1016/j.fsi.2024.109595.
115. S. A. M. Ali, M. Prattipati Geya Sai, and B. Hameeda, "Downregulation of biofilm genes (csgD and bcsA) in Salmonella enterica by metalloprotease of B. subtilis MAH84," *Food Bioscience,* vol. 58, p. 103665, 2024/04/01/ 2024, doi: https://doi.org/10.1016/j.fbio.2024.103665.
116. S. D. Shah, H. Patel, S. M. Saiyad, and B. Bajpai, "Effect of a phthalate derivative purified from Bacillus zhangzhouensis SK4 on quorum sensing regulated virulence factors of Pseudomonas aeruginosa," *Microbial Pathogenesis,* vol. 191, p. 106664, 2024/06/01/ 2024, doi: https://doi.org/10.1016/j.micpath.2024.106664.
117. F. Li *et al.*, "Effects of dietary Bacillus cereus supplementation on the growth performance, serum physiology and biochemistry, Nrf2, TLR/NF-κB signaling pathways, and intestinal health of juvenile coho salmon (Oncorhynchus kisutch)," *Aquaculture Reports,* vol. 36, p. 102177, 2024/06/01/ 2024, doi: https://doi.org/10.1016/j.aqrep.2024.102177.
118. G. Xie *et al.*, "Functional genomic characterization unveils probiotic features of Bacillus cereus G1-11 isolated from the gut of the hybrid grouper (Epinephelus fuscoguttatus♀ × E. lanceolatus♂)," *LWT,* vol. 184, p. 115088, 2023/07/15/ 2023, doi: https://doi.org/10.1016/j.lwt.2023.115088.

119. G. Xie *et al.*, "Effects of Dietary Multi-Strain Probiotics on Growth Performance, Antioxidant Status, Immune Response, and Intestinal Microbiota of Hybrid Groupers (Epinephelus fuscoguttatus female symbol x E. lanceolatus male symbol)," *Microorganisms,* vol. 12, no. 7, Jul 2 2024, doi: 10.3390/microorganisms12071358.
120. M.-Q. Zhang *et al.*, "The dietary supplementation with probiotic Bacillus cereus G1-11 improved the growth parameters, immune performances, intestinal histomorphology, and disease resistance of hybrid grouper (Epinephelus fuscoguttatus ♀ × Epinephelus lanceolatus ♂)," *Animal Feed Science and Technology,* vol. 329, p. 116495, 2025/11/01/ 2025, doi: https://doi.org/10.1016/j.anifeedsci.2025.116495.
121. Y. Feng *et al.*, "Combined analysis of 16S rRNA sequencing and metabolomics reveals the growth-promoting mechanism of compound probiotics in zig-zag eel (Mastacembelus armatus)," *Aquaculture Reports,* vol. 40, p. 102571, 2025/03/15/ 2025, doi: https://doi.org/10.1016/j.aqrep.2024.102571.
122. J. Cadangin *et al.*, "Effects of dietary supplementation of Bacillus, β-glucooligosaccharide and their synbiotic on the growth, digestion, immunity, and gut microbiota profile of abalone, Haliotis discus hannai," *Aquaculture Reports,* vol. 35, p. 102027, 2024/04/01/ 2024, doi: https://doi.org/10.1016/j.aqrep.2024.102027.
123. W. J. Jang *et al.*, "Characterization of a Bacillus sp. KRF-7 isolated from the intestine of rockfish and effects of dietary supplementation with mannan oligosaccharide in rockfish aquaculture," *Fish Shellfish Immunol,* vol. 119, pp. 182-192, Dec 2021, doi: 10.1016/j.fsi.2021.09.039.
124. W. J. Jang *et al.*, "Characteristics and biological control functions of Bacillus sp. PM8313 as a host-associated probiotic in red sea bream (Pagrus major) aquaculture," *Anim Nutr,* vol. 12, pp. 20-31, Mar 2023, doi: 10.1016/j.aninu.2022.08.011.
125. V. Kemsawasd *et al.*, "Efficient recovery of functional biomolecules from shrimp (Litopenaeus vannamei) processing waste for food and health applications via a successive co-culture fermentation approach," *Current Research in Food Science,* vol. 9, p. 100850, 2024/01/01/ 2024, doi: https://doi.org/10.1016/j.crfs.2024.100850.
126. W. Yang, M. Wang, H. Wang, C. Zhang, Q. Zhang, and H. Xiao, "Exploitation of the biocontrol potential of a marine-derived Bacillus velezensis and its application on postharvest strawberry," *Food Control,* Article vol. 161, 2024, Art no. 110311, doi: 10.1016/j.foodcont.2024.110311.
127. A. Anitha, V. J. R. Kumar, J. C. Anjana, M. P. Prabhakaran, and P. G. Preena, "Exploring the microbial diversity of zoanthids: a gateway to novel marine natural products and biotechnological breakthroughs," *Biologia,* vol. 80, no. 2, pp. 369-388, 2024, doi: 10.1007/s11756-024-01846-8.
128. P. Kumari, S. Mane, A. Singh, K. Chauhan, and N. K. Taneja, "Green technologies for production of microbial bioplastics from agricultural biowaste: a review," *Biomass Conversion and Biorefinery,* vol. 15, no. 9, pp. 13143-13163, 2024, doi: 10.1007/s13399-024-06249-y.

129. F. Jafarzadeh, L. Roomiani, M. C. Dezfoulnejad, M. J. Baboli, and A. A. Sary, "Harnessing paraprobiotics and postbiotics for enhanced immune function in Asian seabass (Lates calcarifer): Insights into pattern recognition receptor signaling," *Fish & Shellfish Immunology,* vol. 151, p. 109725, 2024/08/01/ 2024, doi: https://doi.org/10.1016/j.fsi.2024.109725.
130. S. Bharathi *et al.*, "Harnessing the potential of marine bacteria: Production, kinetics, and characterization of bacterial nanocellulose," *Biocatalysis and Agricultural Biotechnology,* vol. 58, 2024, doi: 10.1016/j.bcab.2024.103156.
131. G. Yu *et al.*, "Host-associated Bacillus velezensis T20 improved disease resistance and intestinal health of juvenile turbot (Scophthalmus maximus)," *Aquaculture Reports,* vol. 35, p. 101927, 2024/04/01/ 2024, doi: https://doi.org/10.1016/j.aqrep.2024.101927.
132. K. Amoah *et al.*, "Identification and characterization of four Bacillus species from the intestine of hybrid grouper (Epinephelus fuscoguttatus♀ × E. lanceolatus♂), their antagonistic role on common pathogenic bacteria, and effects on intestinal health," *Fish & Shellfish Immunology,* vol. 152, p. 109795, 2024/09/01/ 2024, doi: https://doi.org/10.1016/j.fsi.2024.109795.
133. B. Tripathy, P. P. Sahoo, H. Sundaray, and A. P. Das, "In-vitro biodegradation of discarded marine microplastics across the eastern coast of the Bay of Bengal, India using Exiguobacterium sp," *Environmental Chemistry and Ecotoxicology,* vol. 6, pp. 236-247, 2024, doi: 10.1016/j.enceco.2024.07.003.
134. D. Saiyam, A. Dubey, M. A. Malla, and A. Kumar, "Lipopeptides from Bacillus: unveiling biotechnological prospects-sources, properties, and diverse applications," *Braz J Microbiol,* vol. 55, no. 1, pp. 281-295, Mar 2024, doi: 10.1007/s42770-023-01228-3.
135. Y. Xu *et al.*, "Macrolactin XY, a Macrolactin Antibiotic from Marine-Derived Bacillus subtilis sp. 18," *Marine Drugs,* Article vol. 22, no. 8, 2024, Art no. 331, doi: 10.3390/md22080331.
136. R. Sultana and I. Joseph, "Marine protease-producing bacteria as potential probionts for advancing mariculture," *Biocatalysis and Agricultural Biotechnology,* vol. 61, p. 103392, 2024/10/01/ 2024, doi: https://doi.org/10.1016/j.bcab.2024.103392.
137. F. Rangel *et al.*, "Novel chitinolytic Bacillus spp. increase feed efficiency, feed digestibility, and survivability to Vibrio anguillarum in European seabass fed with diets containing Hermetia illucens larvae meal," *Aquaculture,* vol. 579, p. 740258, 2024/01/30/ 2024, doi: https://doi.org/10.1016/j.aquaculture.2023.740258.
138. F. Rangel *et al.*, "Isolation of Chitinolytic Bacteria from European Sea Bass Gut Microbiota Fed Diets with Distinct Insect Meals," *Biology (Basel),* vol. 11, no. 7, Jun 25 2022, doi: 10.3390/biology11070964.
139. S. Asharaf, K. Chakraborty, S. K. Paulose, S. Dhara, R. D. Chakraborty, and C. Varghese, "Photoprotective sulfated mannogalactan from heterotrophic Bacillus velezensis blocks UV-A mediated matrix metalloproteinase expression and nuclear DNA damage in human dermal fibroblast," *J Photochem Photobiol B,* vol. 260, p. 113022, Nov 2024, doi: 10.1016/j.jphotobiol.2024.113022.
140. B.-Y. Zhang *et al.*, "Preventive and reparative potentials of heat-inactivated and viable commensal Bacillus pumilus SE5 in ameliorating the adverse impacts of high soybean meal in grouper (Epinephelus coioides)," *Fish & Shellfish Immunology,* vol. 153, p. 109846, 2024/10/01/ 2024, doi: https://doi.org/10.1016/j.fsi.2024.109846.

141. Y.-Z. Sun, H.-L. Yang, K.-P. Huang, J.-D. Ye, and C.-X. Zhang, "Application of autochthonous Bacillus bioencapsulated in copepod to grouper Epinephelus coioides larvae," *Aquaculture,* vol. 392-395, pp. 44-50, 2013/05/10/ 2013, doi: https://doi.org/10.1016/j.aquaculture.2013.01.037.
142. R. K. Govindarajan *et al.*, "Purification and structural analysis of tannase from novel bacteria of Bacillus cereus strain KMS3-1 isolated in marine sediment," *Food Bioscience,* vol. 61, 2024, doi: 10.1016/j.fbio.2024.104664.
143. S. Ts, M. G, K. K. G, P. Ragothaman, R. K. Velu, and S. P, "Secondary metabolite profiling using HR-LCMS, antioxidant and anticancer activity of Bacillus cereus PSMS6 methanolic extract: In silico and in vitro study," *Biotechnol Rep (Amst),* vol. 42, p. e00842, Jun 2024, doi: 10.1016/j.btre.2024.e00842.
144. L. Xiang *et al.*, "Study on the effects and mechanisms of the antagonistic bacterium Bacillus subtilis JSHY-K3 against Vibrio parahaemolyticus causing acute hepatopancreatic necrosis disease in shrimp Penaeus vannamei," *Aquaculture Reports,* vol. 37, p. 102254, 2024/08/01/ 2024, doi: https://doi.org/10.1016/j.aqrep.2024.102254.
145. Y. Gao, R. Tan, Z. Wang, L. Qiang, and H. Yao, "The effects of Bacillus subtilis on the immunity, mucosal tissue morphology, immune-related gene transcriptions, and intestinal microbiota in flounder (Paralichthys olivaceus) with two feeding methods: Continuous versus discontinuous feeding," *Veterinary Immunology and Immunopathology,* vol. 271, p. 110742, 2024/05/01/ 2024, doi: https://doi.org/10.1016/j.vetimm.2024.110742.
146. R. Okamura *et al.*, "The new seriniquinone glycoside by biological transformation using the deep sea-derived bacterium Bacillus licheniformis KDM612," *Journal of Antibiotics,* Article vol. 77, no. 8, pp. 515-521, 2024, doi: 10.1038/s41429-024-00729-z.
147. N. P. Arslan *et al.*, "A review on bacteria-derived antioxidant metabolites: their production, purification, characterization, potential applications, and limitations," *Arch Pharm Res,* vol. 48, no. 4, pp. 253-292, Apr 2025, doi: 10.1007/s12272-025-01541-5.
148. S. Asharaf, K. Chakraborty, C. Varghese, R. D. Chakraborty, S. K. Paulose, and S. Dhara, "A sulfated fucopyranosyl-glucosamino-glucopyranose from marine heterotrophic Bacillus tequilensis regulates inflammatory cytokines in lipopolysaccharide-stimulated human monocyte THP-1 cells," *International Journal of Biological Macromolecules,* vol. 314, p. 144053, 2025/06/01/ 2025, doi: https://doi.org/10.1016/j.ijbiomac.2025.144053.
149. N. Deng *et al.*, "Agricultural residue-based Bacillus tequilensis Bt-RS fermentation enhances banana growth, root metabolism, and rhizosphere microbiome," *Scientia Horticulturae,* vol. 352, p. 114404, 2025/10/01/ 2025, doi: https://doi.org/10.1016/j.scienta.2025.114404.
150. C. Celesti *et al.*, "Bacteria derived bioactive compounds: A valuable tool for the electrochemical detection of arsenic (III) ions in contaminated water," *Sensors and Actuators Reports,* vol. 10, p. 100349, 2025/12/01/ 2025, doi: https://doi.org/10.1016/j.snr.2025.100349.
151. F. R. P. A. Padayao *et al.*, "Bioactivity screening and chemical profiling of Halymenia durvillei fermented by indigenous seaweed-associated Bacillus species," *The Microbe,* vol. 8, p. 100491, 2025/09/01/ 2025, doi: https://doi.org/10.1016/j.microb.2025.100491.

152. P. Vasudhevan, D. Arul, H. M. Lo, S. Singh, and D. Varshney, "Biorefinery-based production of marine-derived alkaline protease from Bacillus subtilis: A promising biocatalyst for breast cancer treatment and detergent formulation applications," *Biomass and Bioenergy,* Article vol. 202, 2025, Art no. 108198, doi: 10.1016/j.biombioe.2025.108198.
153. M. Guo *et al.*, "Effects of dietary Bacillus pumilus on the growth, intestinal health, lipid metabolism, and mTOR signaling pathway of juvenile coho salmon (Oncorhynchus kisutch)," *Water Biology and Security,* vol. 4, no. 1, p. 100313, 2025/01/01/ 2025, doi: https://doi.org/10.1016/j.watbs.2024.100313.
154. X. Wang *et al.*, "Enhanced biodegradation of crude oil by phosphate-solubilizing bacteria Bacillus subtilis PSB-1: Overcoming soluble phosphorus deficiency," *Journal of Environmental Management,* vol. 391, p. 126426, 2025/09/01/ 2025, doi: https://doi.org/10.1016/j.jenvman.2025.126426.
155. R. Thakor *et al.*, "Enhanced Synthesis, Purification, and Characterization of a Marine Bacterial Consortium-Derived Protease Enzyme With Destaining and Keratinolytic Activity," *Biotechnology and Applied Biochemistry,* Article 2025, doi: 10.1002/bab.2711.
156. S. Ma *et al.*, "Epimeric Macrolactin Analogs From a Marine-Derived Bacillus velezensis Against Herpesvirus," *Chemistry and Biodiversity,* Article 2025, doi: 10.1002/cbdv.202402748.
157. S. Yan *et al.*, "Evaluating Rhodopseudomonas palustris, Saccharomyces cerevisiae, and Bacillus licheniformis as substitutes for microalgae food source: Impacts on growth, survival, gut microbiota, and nutrition of Cyclina sinensis," *Aquaculture Reports,* vol. 42, p. 102839, 2025/07/15/ 2025, doi: https://doi.org/10.1016/j.aqrep.2025.102839.
158. G. Qin *et al.*, "Evaluation of probiotic properties and safety of a Bacillus strain for shrimp farming: Integrating in vitro testing, genomic analysis and in vivo validation," *Microbiological Research,* vol. 297, p. 128179, 2025/08/01/ 2025, doi: https://doi.org/10.1016/j.micres.2025.128179.
159. S. Sonbhadra and L. M. Pandey, "Exploring marine and psychrophilic bacteria for biosurfactant production and oil spill mitigation," *Bioresource Technology Reports,* vol. 32, p. 102303, 2025/12/01/ 2025, doi: https://doi.org/10.1016/j.biteb.2025.102303.
160. A. Viswan *et al.*, "Exploring the bioactivity of Bacillus siamensis isolated from marine sediments," *Discover Applied Sciences,* Article vol. 7, no. 7, 2025, Art no. 662, doi: 10.1007/s42452-025-07279-5.
161. R. Liu *et al.*, "Heterologous expression, enzymatic properties, product analysis and molecular docking of assimilative nitrite reductase (NiR) in Bacillus velezensis GXMZU-B1 derived from mariculture," *International Journal of Biological Macromolecules,* vol. 291, p. 139047, 2025/02/01/ 2025, doi: https://doi.org/10.1016/j.ijbiomac.2024.139047.
162. H. Ma *et al.*, "Identification and characterization of a novel bacteriocin PCM7-4 and its antimicrobial activity against Listeria monocytogenes," *Microbiological Research,* vol. 290, p. 127980, 2025/01/01/ 2025, doi: https://doi.org/10.1016/j.micres.2024.127980.
163. X.-K. Zhu *et al.*, "Isolation and characterization of a marine strain: Bacillus inaquosorum, and its feeding effects on Sebastes schlegelii as feed additive," *Aquaculture,* vol. 606, p. 742556, 2025/08/15/ 2025, doi: https://doi.org/10.1016/j.aquaculture.2025.742556.
164. R. S. R. Gayathri *et al.*, "Isolation, identification and bioprospecting potential of Bacillus subtilis, endophytic bacterium from Bruguiera gymnorrhiza (L.) Lam. ex Savigny," *Microb Pathog,* vol. 203, p. 107458, Jun 2025, doi: 10.1016/j.micpath.2025.107458.

165. C. Lv, Q. Kong, Z. Yu, W. Feng, K. Zhao, and Z. Sun, "Medium optimization of three marine probiotics and their application in the culture of Sebastes schlegelii," *Microbial Pathogenesis,* vol. 204, p. 107599, 2025/07/01/ 2025, doi: https://doi.org/10.1016/j.micpath.2025.107599.
166. J. Jayaprakash *et al.*, "Molecular characterization and molecular docking of xylanase produced by novel species Bacillus aryabhattai isolated from mangrove soil and its biodegradation efficiency," *Biocatalysis and Agricultural Biotechnology,* vol. 66, 2025, doi: 10.1016/j.bcab.2025.103582.
167. H. Wang, Y. Wang, Y. Li, G. Wang, T. Shi, and B. Wang, "New Pyridinium Compound from Marine Sediment-Derived Bacterium Bacillus licheniformis S-1," *Molecules,* Article vol. 30, no. 1, 2025, Art no. 7, doi: 10.3390/molecules30010007.
168. S. O. Santos *et al.*, "Optimizing difenoconazole degradation via sequential photoelectrochemical oxidation and biodegradation treatments," *Chemosphere,* vol. 380, p. 144464, Jun 2025, doi: 10.1016/j.chemosphere.2025.144464.
169. C. Prashar *et al.*, "Potent targeted larvicidal activities of marine-derived Bacillus sp. bacterial extracts on mosquito vectors," *Scientific Reports,* Article vol. 15, no. 1, 2025, Art no. 8094, doi: 10.1038/s41598-024-80777-5.
170. J. Wang, F. Li, W. Li, Y. Li, J. Zhang, and S. Qin, "Progress in Preparation Technology and Functional Research On Marine Bioactive Peptides," *Mar Biotechnol (NY),* vol. 27, no. 1, p. 42, Feb 5 2025, doi: 10.1007/s10126-024-10401-0.
171. L. Liao, J. Song, R. Qiu, C. Liu, T. Li, and L. Pan, "Synthetic microbial communities for simultaneous nitrogen removal and pathogen control: probiotic isolation, efficacy analysis, consortium construction and application potential," *Journal of Water Process Engineering,* vol. 77, p. 108400, 2025/09/01/ 2025, doi: https://doi.org/10.1016/j.jwpe.2025.108400.
172. H. Wang *et al.*, "Unlocking the chemical basis of fermented golden pompano (Trachinotus ovatus) inoculated with indigenous Bacillus subtilis: Focus on the role of lipid oxidation on volatile flavor formation," *Food Chemistry,* vol. 472, p. 142929, 2025/04/30/ 2025, doi: https://doi.org/10.1016/j.foodchem.2025.142929.
173. X. Cai *et al.*, "Whole genome sequencing and metabonomics analysis reveal the biodegradation process of deoxynivalenol in a safety Bacillus velezensis T-3 isolated from the gut of sea cucumber," *Food Chemistry: X,* vol. 31, p. 103190, 2025/10/01/ 2025, doi: https://doi.org/10.1016/j.fochx.2025.103190.
174. M. Gustilatov *et al.*, "The effect of biofloc density and Bacillus sp. NP5 supplementation on bacterial inhibition, antibiofilm activity, and the immunity of the Pacific white shrimp (Penaeus vannamei) against Vibrio parahaemolyticus," *Comparative Immunology Reports,* vol. 8, p. 200228, 2025/06/01/ 2025, doi: https://doi.org/10.1016/j.cirep.2025.200228.
175. J.-Y. Lee *et al.*, "Antimicrobial and immunoprotective effects of Bacillus safensis BS22LVI against acute hepatopancreatic necrosis disease in whiteleg shrimp (Penaeus vannamei)," *Aquaculture,* vol. 613, p. 743327, 2026/02/01/ 2026, doi: https://doi.org/10.1016/j.aquaculture.2025.743327.
176. Y.-Y. Liang and C.-H. Liu, "Integrative effects of Bacillus tropicus FG2 on growth performance, immunity, gut microbiota, and metabolome in short-finned eel, Anguilla bicolor pacifica," *Fish & Shellfish Immunology,* vol. 168, p. 110925, 2026/01/01/ 2026, doi: https://doi.org/10.1016/j.fsi.2025.110925.

177. X. Ge *et al.*, "The versatility of Bacillus tequilensis Bt-CO as an additive: Antagonizing pathogens, enhancing immunity, promoting intestinal health, and optimizing gut microbiota of tilapia," *Animal Nutrition,* 2025/08/26/ 2025, doi: https://doi.org/10.1016/j.aninu.2025.07.001.
178. J. Huang *et al.*, "Probiotic Bacillus tequilensis protected Micropterus salmoides against Aeromonas veronii infection by modulating digestion, immunity, and intestinal microbiota and metabolites," *Aquaculture,* vol. 610, p. 742943, 2026/01/01/ 2026, doi: https://doi.org/10.1016/j.aquaculture.2025.742943.
179. E. S. Bindiya, K. J. Tina, S. S. Raghul, and S. G. Bhat, "Characterization of Deep Sea Fish Gut Bacteria with Antagonistic Potential, from Centroscyllium fabricii (Deep Sea Shark)," *Probiotics Antimicrob Proteins,* vol. 7, no. 2, pp. 157-63, Jun 2015, doi: 10.1007/s12602-015-9190-x.
180. B. E.S. and S. G. Bhat, "Bacteriocins BaCf3 and BpSl14 with anticancer and antibiofilm potential from probiotic Bacillus amyloliquefaciens BTSS3 and Bacillus pumilus SDG14 isolated from gut of marine fishes: Enhanced production, Purification and Characterization," Cochin University of Science and Technology, Cochin, Kerala, 2017.
181. F. Abasolo-Pacheco *et al.*, "Isolation and use of beneficial microbiota from the digestive tract of lions-paw scallopNodipecten subnodosusand winged pearl oysterPteria sternain oyster aquaculture," *Aquaculture Research,* vol. 47, no. 10, pp. 3042-3051, 2016, doi: 10.1111/are.12754.
182. R. Escamilla-Montes *et al.*, "Isolation and Characterization of Potential Probiotic Bacteria Suitable for Mollusk Larvae Cultures," *The Thai Journal of Veterinary Medicine,* vol. 45, no. 1, pp. 11-21, 2015, doi: 10.56808/2985-1130.2614.
183. H. Fei *et al.*, "Effects of Bacillus amyloliquefaciens and Yarrowia lipolytica lipase 2 on immunology and growth performance of Hybrid sturgeon," *Fish Shellfish Immunol,* vol. 82, pp. 250-257, Nov 2018, doi: 10.1016/j.fsi.2018.08.031.
184. J. Wu *et al.*, "Isolation and characterization of Bacillus sp. GFP-2, a novel Bacillus strain with antimicrobial activities, from Whitespotted bamboo shark intestine," *AMB Express,* vol. 8, no. 1, p. 84, May 22 2018, doi: 10.1186/s13568-018-0614-3.
185. R. Ghanei-Motlagh *et al.*, "Quorum Quenching Properties and Probiotic Potentials of Intestinal Associated Bacteria in Asian Sea Bass Lates calcarifer," *Mar Drugs,* vol. 18, no. 1, Dec 26 2019, doi: 10.3390/md18010023.
186. Y. Z. Sun, H. L. Yang, R. L. Ma, and W. Y. Lin, "Probiotic applications of two dominant gut Bacillus strains with antagonistic activity improved the growth performance and immune responses of grouper Epinephelus coioides," *Fish Shellfish Immunol,* vol. 29, no. 5, pp. 803-9, Nov 2010, doi: 10.1016/j.fsi.2010.07.018.
187. A. T. Wahyudi, J. A. Priyanto, W. Maharsiwi, and R. I. Astuti, "Screening and Characterization of Sponge-Associated Bacteria Producing Bioactive Compounds Anti-Vibrio sp," *American Journal of Biochemistry and Biotechnology,* vol. 14, no. 3, pp. 221-229, 2018, doi: 10.3844/ajbbsp.2018.221.229.
188. R. A. Santos *et al.*, "Bacillus spp. Inhibit Edwardsiella tarda Quorum-Sensing and Fish Infection," *Mar Drugs,* vol. 19, no. 11, Oct 23 2021, doi: 10.3390/md19110602.

189. C. R. Serra *et al.*, "Selection of carbohydrate-active probiotics from the gut of carnivorous fish fed plant-based diets," *Sci Rep,* vol. 9, no. 1, p. 6384, Apr 23 2019, doi: 10.1038/s41598-019-42716-7.
190. Y. Y. Liang, S. Y. Hu, and C. H. Liu, "Comparative Analysis of Gut Microbiota Between Fast-Growing and Slow-Growing Short-Finned Eels, Anguilla bicolor pacifica, and the Application of Bacillus tropicus FG2 as a Probiotic to Enhance Growth Performance of Eels," *Animals (Basel),* vol. 16, no. 1, Dec 24 2025, doi: 10.3390/ani16010054.
191. E. P. o. Additives *et al.*, "Guidance on the characterisation of microorganisms used as feed additives or as production organisms," *EFSA J,* vol. 16, no. 3, p. e05206, Mar 2018, doi: 10.2903/j.efsa.2018.5206.
192. A. V. Nair, K. K. Vijayan, K. Chakraborty, and M. Leo Antony, "Diversity and characterization of antagonistic bacteria from tropical estuarine habitats of Cochin, India for fish health management," *World J Microbiol Biotechnol,* vol. 28, no. 7, pp. 2581-92, Jul 2012, doi: 10.1007/s11274-012-1067-5.
193. X. Li *et al.*, "Characterization of a Bacillus velezensis with antibacterial activity and inhibitory effect on common aquatic pathogens," *Aquaculture,* vol. 523, 2020, doi: 10.1016/j.aquaculture.2020.735165.
194. H. L. Yang, Z. Y. Liu, J. T. Jian, J. D. Ye, and Y. Z. Sun, "Host‐associated Bacillus siamensis and Lactococcus petauri improved growth performance, innate immunity, antioxidant activity and ammonia tolerance in juvenile Japanese seabass (Lateolabrax japonicus)," *Aquaculture Nutrition,* vol. 27, no. 6, pp. 2739-2748, 2021, doi: 10.1111/anu.13399.
195. S. Yunzhang, Y. Hongling, L. Zechun, C. Jianbo, and Y. Jidan, "Gut microbiota of fast and slow growing grouper Epinephelus coioides," *African Journal of Microbiology Research,* vol. 3, no. 11, p. 8, 2009.
